# Addressing inequity in palliative care provision for older people living with multimorbidity. Perspectives of community-dwelling older people on their palliative care needs: A scoping review

**DOI:** 10.1177/02692163221118230

**Published:** 2022-08-24

**Authors:** Caroline Jane Nicholson, Sarah Combes, Freda Mold, Helen King, Richard Green

**Affiliations:** 1 University of Surrey, Guildford, UK; 2 St Christopher’s Hospice, London, UK

**Keywords:** Aged, multimorbidity, frailty, terminal care, needs assessment

## Abstract

**Background:**

Older people living with multimorbidity are projected to become the main recipients of palliative care in the coming decades, yet there is limited evidence regarding their expressed palliative care needs to inform person-centred care.

**Aim:**

To understand the palliative care needs of community-dwelling people aged ⩾60 living with multimorbidity in the last 2 years of life.

**Design:**

A scoping review following Arksey and O’Malley.

**Data sources:**

Three international electronic databases (CINAHL, Ovid Medline, PsycINFO) were searched from March 2018 to December 2021. Reference lists were hand searched. Eligible papers were those reporting empirical data on older people’s needs.

**Results:**

From 985 potential papers, 28 studies were included, published between 2002 and 2020; sixteen quantitative, nine qualitative and three mixed methods. Data were extracted and presented under the holistic palliative care domains of need: physical, psychological, social, spiritual, and additionally practical needs. Different measurement tools (*n* = 29) were used, of which 20 were multidimensional. Primacy in reporting was given to physical needs, most commonly pain and function. Social and practical needs were often prioritised by older people themselves, including maintaining social connections and accessing and receiving individualised care.

**Conclusion:**

Identifying the palliative care needs that matter most to older people with multimorbidity requires the recognition of their concerns, as well as their symptoms, across a continuum of living and dying. Available evidence is superficial. Supporting end of life provision for this growing and underserved population necessitates a shift to tailored multidimensional tools and community focussed integrated care services.

**What is already known about this topic?**

- Multimorbidity is increasing substantially worldwide, is associated with greater use of healthcare services, lower quality and quantity of life, and rises with age.
- Older people with multimorbidity are expected to become the main recipients of palliative care in the coming decades; however, there is limited evidence of their specific needs.
- Older people’s voices are vital to understanding their own palliative care needs and priorities, but these voices are hampered by structural inequities in service provision.

**What this paper adds?**

- This is the first paper reporting on the expressed palliative care needs of community-dwelling older people with multimorbidity.
- The most common palliative care needs identified across need domains were pain, function, unhappiness, staying socially connected, future planning, person-centred care and having meaning and purpose in life.
- This paper highlights different priorities between the reported items in tools used to collect palliative care need and needs expressed by older people with multimorbidity.

**Implications for practice, theory or policy?**

- Further evidence is required to understand need to support service changes required to provide accessible, person-centred care to this underserved population.
- Multidimensional palliative care tools require refining to encompass complexity beyond the standard domains of palliative care.
- Community palliative care provision should involve the integration of care across sectors and recognise the diversity of needs across the continuum of living and dying well for older people with multimorbidity.

## Introduction

Multimorbidity is defined as the co-occurrence of at least two long-term conditions in the same individual.^1^ Multimorbidity is associated with increased mortality, lower quality of life, and greater use of healthcare services, including unplanned hospital admissions. Globally multimorbidity is increasing, driven in part by worldwide ageing.^2^ Predisposing factors include socio-economic status, certain ethnic backgrounds, being female, and the co-existence of physical and mental health needs.^3^ Prevalence estimates vary, ranging from 13% to 72% of the population.^2^ Recent estimates in England suggest more than half of people aged 65 and above^4^ are living with multimorbidity. Older people living with multimorbidity are projected to become the main recipients of palliative care in the coming decades.^5^ However, little is known about their palliative care needs and they are currently underserved by palliative care services for diverse reasons (1) frailty and non-malignant conditions contribute to an unpredictable dying trajectory that makes identifying palliative care needs and the start of the end-of-life phase challenging, (2) services have historically been single disease focussed,^6,7^ (3) age related structural inequalities impede access to care.^8^

This paper focusses on the perceived palliative care needs of community-dwelling older people living with multimorbidity, and their proxies. Most people will live out their last years of life in the community, requiring integrated care addressing social as well as health needs. This paper also includes studies of those who transition between acute and community settings, a common experience for community-dwelling older people as they near the end of life. Within this review Bradshaw’s taxonomy of need, either an ‘expressed’ or ‘normative’ need, is utilised.^9,10^ An expressed need is ‘what an individual demands’, defined in this paper as what an older person themselves articulates as a need, while a normative need is ‘what a professional or family members think an individual wants’.^10^ Palliative care need is often conceptualised through four domains: physical, psychological, social and spiritual.^11^ We also included a fifth domain, practical need, reported by Larkin and Hegarty as important for capturing essential aspects of managing daily living (e.g. finances).^12^ Definitions for these five domains (Table 1) were agreed by the authors and a clinical reference group of palliative and geriatric clinicians.

**Table 1.** Definitions of need by palliative care domain^11,12^.

Need domain	Definition
Physical	Any need arising from a biological cause related to symptom burden or treatment, including decline in physical function and core activities related to activities of daily living.
Social	Any need relating to social roles and/or functioning. Social needs include: • *Relational care needs*: related to being in connection with others, including family carers and care professionals. • *Social network needs*: related to both supporting and being supported by family, friends, and neighbourhoods.
Psychological	Any need arising from psychological symptoms relating to present experience(s), including anxiety, depression, health, self-esteem, self-worth, and adjustment to a situation.
Spiritual	Any need relating to questions or thoughts about the nature of existence and being. This includes but is broader than religious needs. Spiritual needs include: • Needs that arise from distress or questioning the loss of future or hope. • Needs that relate to the way a person seeks and expresses their connectedness to existence.
Practical	Any need related to a requirement for resource(s) to enable a person to manipulate their environment so that they can live as well as possible both now and in the future. Practical needs include: • Environmental care needs, such as housing, aids, or adaptations. • Informational and/or financial needs. • Care planning, including planning for the future. • Access to and receipt of individualised care.

## Methods

### Research question

This review asks: what are the perspectives of community-dwelling older people living with multimorbidity, and their proxies, on their palliative care needs?

### Design

Arksey and O’Malley’s^13^ scoping review method was selected. A scoping review is a form of research synthesis that allows literature in a particular area to be mapped, to identify the types of evidence available, gaps in the literature, and key concepts. A scoping review also permits collation and analysis of data from heterogeneous literature and post hoc development of inclusion/exclusion criteria,^14^ to get an initial understanding of the breadth of the topic before restricting focus. This was important for a topic that has historically received limited research attention, where the evidence base has a dearth of literature indexed to multimorbidity, and where key search terms can be used differently across literature, making the identification of relevant evidence difficult (e.g. the conflation of multimorbidity and comorbidity, or synonymous terms, contributing to identifying papers focussing on single conditions).

### Inclusion and exclusion criteria

Due to the lack of papers indexed as multimorbidity studies, we needed to keep our inclusion criteria broad (Table 2; see Supplemental Appendix 1 for more detailed information). Papers were included where a distinct, disaggregated sub-sample of older people living with multiple conditions were included within a larger total sample for a study among other populations for comparison. Papers that solely referred to a single disease, e.g. older people living with chronic obstructive pulmonary disease, were excluded. Papers were included where participants were living with two or more conditions, as per NICE guidance.^15^

**Table 2.** Inclusion criteria.

Category	Inclusion
Older People	More than half the sample is 60 years of age or older. This includes formal and informal caregivers of people aged 60 years of age or older. (Unless relevant information is unavailable)
Multi-morbidity	More than half of the sample are older people with two or more chronic conditions, one of which may be author-identified frailty, this includes carers of the above; OR Where information on the number and type of conditions is available, multimorbidity is explicitly and relevantly addressed in the analysis and presentation of findings (Unless relevant information is unavailable)
Palliative and/or End of Life Care	Participants are defined as being in the last 1–2 years of life, or living with advanced disease, or frail (Unless relevant information is unavailable)
Community	The intervention is set in the community, including transitions between community and acute care settings
Study Design	The study presents empirical data
Needs	The study has a focus on needs and unmet needs from an older person’s perspective OR The study has a focus on the needs and unmet needs of older people that is represented by a proxy respondent (e.g. an unpaid carer or care professional)
Relevance	The study is relevant to the research question.

In seeking to examine evidence on end of life needs, palliative and end of life care items were included in the inclusion/exclusion criteria. Palliative care may be required long before the final weeks of life, while NICE include within their end of life definition those living with advanced, progressive, incurable conditions.^16^ Those living with frailty were included as per Smith et al.’s strategy and clinical guidelines for using frailty as a proxy indicator for multimorbidity.^1,15^ However, papers were not excluded if no palliative or end of life information was provided, recognising that end of life can be hard to predict and may not be the primary focus of papers. Few studies were included on this basis, where for most studies limited or no information was available.

Proxies for older people were included with the understanding that older people will sometimes need support to express their needs and to better understand the state of current evidence as to how the needs of older people are currently being collected. However, the focus of the papers must be on older people’s needs, and consequently, papers focussing on professionals’ needs, service organisation, and delivery were excluded.

### Search strategy

The search strategy (Supplemental Appendix 2) was informed by a previous review on the rise of multimorbidity in old age.^1^ The strategy combines both generic terms for multiple, chronic conditions, such as ‘multimorbidity’ or ‘co-morbidity’ (group 1) AND terms for specific diagnoses, such as ‘COPD’, ‘diabetes’ or ‘dementia’ (group 2). This enables retrieval of papers that either use the term multimorbidity or include two or more terms for specific conditions. The list of conditions from Smith et al.’s strategy was adapted as appropriate for this review. Terms were excluded for chronic diseases with early on-set, which are less likely to be found in older populations, as well as terms such as alcohol or substance misuse, which identify specific sub-groups of people whose end of life care needs cannot be assumed to be representative of the broader population. MeSH terms for other concepts included ‘Aged’ (group 3) AND ‘End of Life Care’ or ‘Palliative Care’ (group 4) AND ‘Home-based’ OR ‘Community Health Care Services’ OR ‘Nursing Homes’ OR ‘Social Work’ (group 5). MeSH terms were identified on MEDLINE and adapted for searches on the other databases.

**Figure 1.**
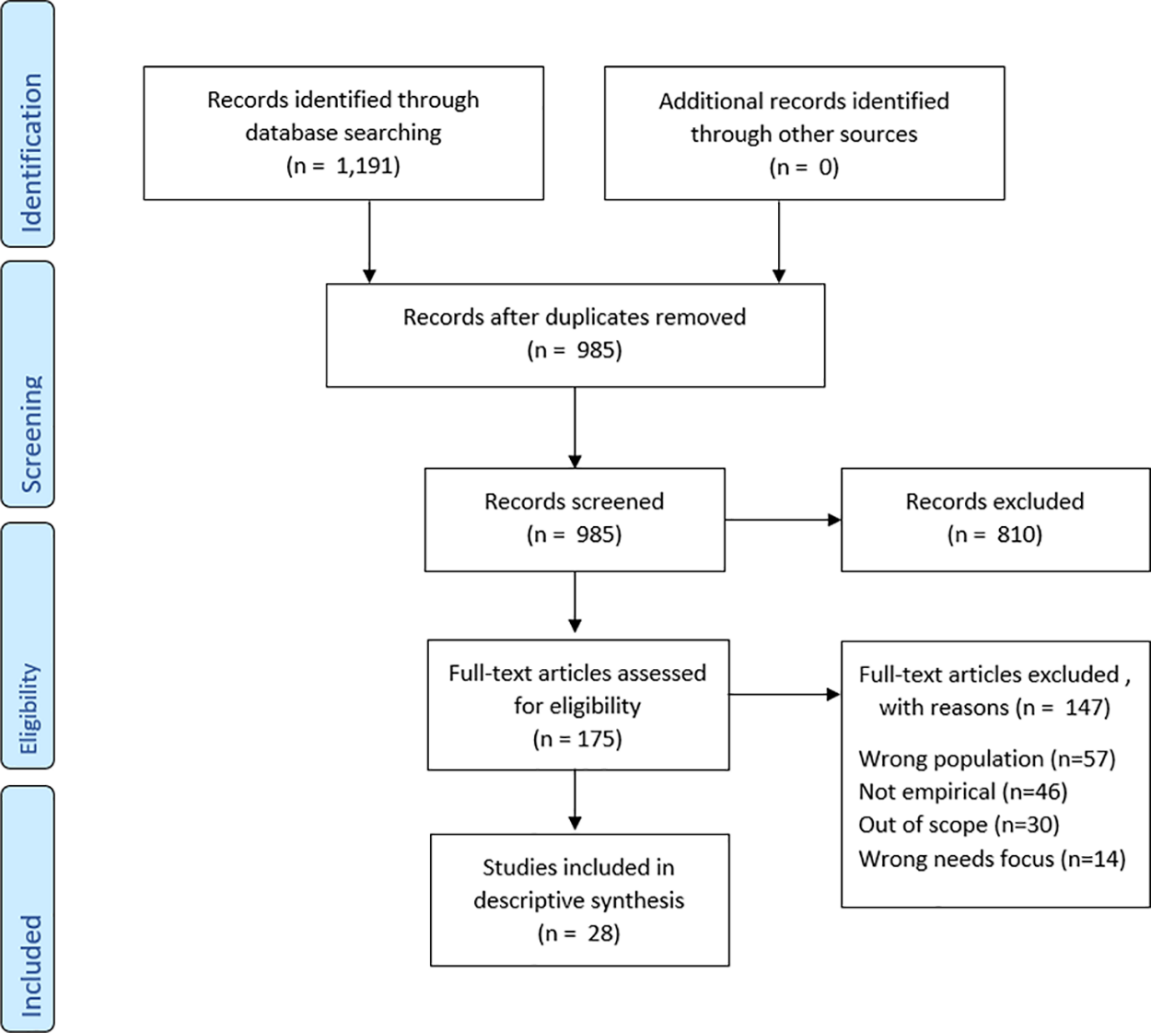
PRISMA flow diagram.^19^

Inclusion and exclusion criteria were calibrated by two team members (CN and SC) independently screening a sample of references (30–100) from each database. Titles and abstracts were screened for each paper by two team members (SC and CN) using inclusion/exclusion criteria (Supplemental Appendix 1) to assess eligibility. Papers that passed screening were reviewed in full and key information from the paper was extracted by the authors. Quality assessment is not required for a scoping review^17^ but the Mixed Methods Appraisal Tool^18^ was used to understand the state of the evidence for included studies (Supplemental Appendix 3). Papers were included on the basis that they addressed the aim of the review rather than based on a quality score. Figure 1 below shows the overall paper selection process.^19^

### Data extraction

Following Arksey and O’Malley,^13^ data were sorted by key themes using the palliative care need domains (Table 1) as a broad analytical framework. Relevant data were recorded in Excel spreadsheets and included author(s), title, year, country, study setting, aim, design, sample, intervention, result(s), measures used, need(s), how the need was reported (self-expressed or by proxy), practice/policy/research implications, and study limitations (Table 3). Within domains, themes and sub-themes were independently identified, then discussed and agreed by two people. Themes are grouped areas of high-level need (e.g. function) and sub-themes are more granular (e.g. ‘activities of daily living’ and ‘mobility’). In the physical domain, a sub-theme of ‘other physical concerns’ collated needs receiving scant attention attributed to hygiene and fatigue (Table 4). Where needs were identified as overlapping palliative domains these were discussed and agreed upon by the authors and clinical reference group covering palliative and geriatric medicine. Greater attention has been given to themes where more evidence has been available across studies. Collated results of the review are shown in the summary of findings related to need (Table 3) and thematic presentation of palliative care needs in the results below (each theme is highlighted in bold).

**Table 3.** Included studies.

Citation and Country	Participants and measurement tools used (including need domains assessed within tools)	Design	Relevant/(Total) Sample	Perspective of Reported Needs	Summary of findings relating to needs
Amblàs-Novellas et al.,^20^ Spain	Patients with a positive NECPAL CCOMS-ICO© tool score, indicating they might benefit from a palliative care approach. Recruited from mixed community settings.	Cross-sectional	377 (782)	Researchers, based on the prevalence of prognostic indicators.	This paper compares those with advanced chronic conditions identified as having palliative care needs across three archetypal end-of-life trajectories: acute (typically cancer), intermittent (typically organ failure) and gradual dwindling (typically dementia or frailty), exploring their physical symptoms. The large number of identified patients with advanced chronic conditions but with no advanced disease criteria reveals that there is a real and not previously well-described cohort of people with advanced frailty and palliative care needs.
Beach et al.,^21^ United States of America	Community-dwelling participants from the National Health and Aging Trends Study (NHATS) with functional limitation for at least one of the following: (Instrumental/) Activities of Daily Living/Mobility	Case control	2645 (4024)	Older person/a proxy if older person unable to respond	High need, high care older adults report much higher levels of adverse consequences from unmet needs for Activities of Daily Living and mobility assistance compared with low need patients, despite more disability compensatory efforts, including environmental modifications, mobility device use, paid caregiver use, and larger informal helper networks.
Bone et al.,^22^ United Kingdom	Decedents of those aged ⩾65 with advanced disease receiving palliative care prior to death in South England (from two postal surveys). EQ5D tool covering Physical (pain), Practical, and Psychological (anxiety) domains.	Survey	96 (688)	Official death registrations and data collected from the decedent’s family member	Those with a primary diagnosis of respiratory disease, and with multiple comorbidities were more likely to have frequent end of life emergency department attendances. Family members revealed concerns around care continuity, confidence in care and support provided, responsiveness of staff to changing need, and untimely discharge with poor coordination leading to readmission.
Brandt et al.,^23^ The Netherlands	Nursing Home (NH) residents with a life expectancy of ⩽6 weeks, or admitted to long term care, recruited from 16 Nursing Homes. The Palliative care Outcome Scale (POS) was used to assess the key domains in the provision of palliative care (all domains). The Global Deterioration Scale was used to assess for dementia.	Cross-sectional	328 (471)	Proxy – Nursing Home Staff	This is the first study that describes the quality of palliative care provided in the last days of the life of nursing home residents who were mainly noncancer patients. The usefulness of the POS tool was evaluated in a NH population including patients with dementia. ‘Non-scores’ for added POS items: ‘not applicable’ and ‘unknown’ were often found in the POS-items patient anxiety, support, life worthwhile, and self-worth. It was also frequently unknown whether the patient’s illness-related personal affairs had been addressed.
Chan and Pang, ^24^ Hong Kong (China)	Cognitively-able long-term care residents aged >65. The Cumulative Illness Rating Scale (CIRS) was used to assess physical needs. The Quality-of-Life Concerns in the End-of-Life Questionnaire (QOLC-E) tool was used to assess across all five need domains.	Cross-sectional	164 (287)	Older People	Findings from the Quality-of-Life Concerns in the End-of-Life Questionnaire (QOLC-E) yielded three positive (value of life, food-related concerns, and care and support) and negative (physical discomfort, negative emotions, and existential distress) subscales. Regarding QOL concerns, members of the frail group were generally less satisfied with their QOL than their non-frail counterparts. The differences were more apparent in the three negative subscales. Considerable numbers were uncertain about their end-of life care preferences, and they preferred their physician to be their surrogate.
Chochinov et al.,^25^ Canada	Frail elderly people, defined as: (1) being over 80 years of age; (2) residing in a personal care home (PCH); and (3) requiring assistance with two or more basic activities of daily living; and (4) having a Cognitive Performance Scale (CPS) of 0–3 (i.e. none to mild cognitive decline). Numerous tools were used: Patient Dignity Inventory (PDI; all domains), Multidimensional Scale of Perceived Social Support (MSPSS; Social, Practical), Revised Edmonton Symptom Assessment Scale (ESAS-R; Physical, Psychological), Structured Interview of Symptoms and Concerns (SISC; Physical, Psychological, Spiritual), Herth Hope Index [HHI] (Spiritual), Spiritual Survey (Spiritual), The Katz Index of Daily Basic Living (Physical), and Hospital Anxiety Depression Scale (HADS; Psychological).	Cross-sectional Part of a prospective, multi-site approach	102 (404)	Participants of the study – including a frail elderly group	The frail elderly group reported the highest prevalence of moderate to extreme desire for death, the lowest level of hope, and the most physical dependencies. Relative to other groups, the frail elderly felt less supported by family, friends or a significant other. While the elderly are not typically described as ‘terminally ill’, 42% of them died over the course of the study. Relative to the other groups, they are the most isolated and report the lowest social support from family, friends or a significant other. They reported feeling the least hope, in the absence of significant worry about the future. Along with reporting the highest desire for death, in the absence of suicidal ideation or disproportionate psychological distress, a picture emerges of elderly residents being at relative ease compared to other study populations. They are not particularly worried or frightened of the future; with an endorsement of desire for death perhaps indicative of a readiness to die.
Gomez-Batiste et al.,^26^ Spain	Patients identified by a doctor or nurse as suffering from at least one advanced chronic condition were recruited from a range of care settings. The NECPAL tool (all domains) was used.	Cross-sectional	785 (1064)	Health professionals	NECPAL+ patients are mainly among the elderly population which is often living at home or in NH. Advanced frailty and dementia are the most common clinical conditions, followed by cancer and organ failure. There are higher proportions of women and non-cancer patients. All the individuals identified would benefit from a palliative care approach.
Goodridge et al.,^27^ Canada	14 registered nurses and eight healthcare aides who had provided care within the last 72 hours before a long-term care resident’s death and four family members who had visited within the same time frame. These interviews relating to 15 resident deaths.	Qualitative interviews	26 (26)	Interviews with decedents’ family members, registered nurses, and health care assistants	Caring behaviours of staff were central to the dying experience and encompassed a wide range of activities. Professional activities, such as assessment and coordination of care, were not readily evident to either families or health care assistants, except in their absence, but were recognised by Research Nurses to be a critical component of end of life (EOL) care. There was little question about the importance of high-quality physical EOL care to family members, registered nurses, and health care assistants. Ensuring a resident was as physically comfortable as possible was the overriding goal of staff, but deficits in dyspnoea management suggest a role for additional staff education. Emotional support of both the resident and family was cited by all participants as key to quality EOL care.
Kayser-Jones,^28^ United States of America	Nursing home residents’ care was observed from when they were identified as being terminally ill until they died. *Findings are drawn from same research undertaken in Kayser-Jones et al. 2003.	Qualitative multi-method (participant observation, in-depth interviews, and event analysis)	189 (189)	Older people, their families, and nursing home staff	Prominent factors that influenced the experience of dying were lack of attention to cultural needs, cognitive status, inadequate staffing, and inappropriate and inadequate communication between health care providers and nursing home residents and their families.
Kayser-Jones et al.,^29^ United States of America	Nursing home residents’ care was observed from when they were identified as being terminally ill until they died. *Findings are drawn from same research undertaken in Kayser-Jones 2002.	Qualitative multi-method (participant observation, in-depth interviews, and event analysis)	189 (189)	Older people, their families, and nursing home staff	The physical environment was not conducive to end-of-life care. Rooms were crowded, there was little privacy, and the facilities were noisy. Often, residents did not receive basic care, such as bathing, oral hygiene, adequate food and fluids, and repositioning. Environmental factors, physical and organisational, influenced end-of-life care. Privacy and space, necessary for residents to have time with their families and bring closure to their lives, emerged as important themes. Inadequate staffing and lack of supervision were critical factors in the care of the residents. In the weeks and months before death, many residents were hungry and thirsty, and their relatives would not be fed. More than half the residents had pressure ulcers.
Kendall et al.,^30^ United Kingdom	All the patients within the 8 synthesised studies included in this paper were living in community settings and were considered to be at risk of dying within the next 12 months by the clinicians who helped enrol them into the studies. The ‘global clinical measure’ tool for frailty (Rockwood et al., 2005; Physical) was used.	Qualitative synthesis (two studies using serial in-depth interviews)	13 [from Lloyd study]; 25 [from Worth study] (440)	Older people, family care-giver, health professionals	Frail, older people, and their family caregivers and service providers all struggled to recall exactly when their health began to deteriorate. Patients focused on staying as well as possible and maintaining autonomy in the face of increasing dependence. They expressed frustration at their declining capacities. Frailty often becomes a way of life, and a new normal and little support is accessed from specialist palliative care services.
Kozlov et al.,^31^ United States of America	Adults aged ⩾60 years living in community settings receiving services at 1 of 5 senior centres located in Brooklyn and Staten Island, New York. Medical comorbidity data were collected using a 22-item medical condition checklist^32^ (Physical). A tool for community-based palliative care screening was also used^33^ (Physical, Social, Practical, Psychological).	Cross-sectional	63 (237)	Older people	The rate of unmet palliative care needs in community-based older adults who attend senior centre events was high and living arrangement and education level were both correlates of unmet palliative care needs. Having less education, higher rates of medical comorbidities and living alone were associated with screening positive for unmet palliative care needs.
Kramer,^34^ United States of America	Elders with advanced chronic disease, eligible for nursing home level care, Medicaid recipients, and considered likely to die within 6 months were selected. Family and care team members for the elder were also participants. Survey reports of the needs addressed by social workers for 120 deceased elders were collected. Five focus groups with interdisciplinary team members were conducted. In-depth interviews with 14 elders and 10 of their family caregivers were also conducted. Author constructed end of life survey items were included, presented in the paper.	Mixed methods (convergent design)	144 (144)	Older people, their family, team members and social workers.	Social workers engaged in efforts to address elder and family caregiver psychological and emotional responses at the end of life and had a high degree of involvement in grief and bereavement, spiritual issues, and funeral planning. Social workers frequently addressed specific psychological issues, grief and bereavement, funeral planning, and spiritual issues with the elders they served.
Kricke et al.,^35^ United States of America	Community-dwelling in the last month of life participants from the National Health and Aging Trends Study (NHATS) who had a proxy who was not an employee of the setting where the individual died. Participants had complete chronic condition data reported before death and received care in the last month of life with a proxy rating the overall quality of that care for the end of life.	Cross-sectional	477 (477)	Proxies of older people with multiple chronic conditions	End of life care domains of coordination, symptom management, shared decision making, respect, and spiritual and emotional support. were assessed for older adults living with multiple chronic conditions (MCC). A little over half of decedents’ proxies rated overall end of life care quality as ‘excellent’, suggesting a need to improve care for dying older adults with MCC. All domains listed, except symptom management, mattered when rating overall end-of-life care quality for older adults with MCC Findings suggest that dying older adults with MCC may prioritise psychosocial over physiological needs.
Lambotte et al.,^36^ Belgium	Community-dwelling older adults aged ⩾60 years in need of assistance who both did and did not receive and received no care or support. The Comprehensive Frailty Assessment Instrument (CFAI; Physical, Psychological, Social, Practical).	Cross-sectional	12,481 (12,481)	Older people	Significant differences were found when comparing the different combinations of care use to frailty levels in community-dwelling older adults in need of assistance. Older adults who were more likely to receive care from family (both nuclear and extended) in combination with care from all types of formal care providers had higher proportions of psychological frailty and the highest proportions of physical frailty. Older adults in need of assistance who were more likely to receive care only from all types of formal care providers had higher proportions of physical frailty and were even most psychologically frail. Older adults in need of assistance who were more likely to receive informal care from nuclear family only were more socially frail than expected, while those receiving little or no care from different types of informal caregivers had higher proportions of social frailty.
Lee et al.,^37^ South Korea	Community-dwelling aged ⩾40 people received continuous home care nursing throughout the stable and final death stages and diagnosed with a non-cancerous disease.	Survey	115 (115)	Home healthcare nurse specialists	The care needs of ‘coordination among family or relatives’ and ‘support for fundamental need’ were more important in the stable stage prior to death than in the near-death stage. ‘Loss, grief care’ was more important in the near-death stage than in the stable stage. The care need ‘physical symptoms management’ was the most difficult to meet in both stages. A lower Palliative Performance Scale score was associated with a higher level of care need, particularly in the ‘management of physical symptoms’ and ‘psychological support’ realms in the stable stage and in the ‘coordination among family or relatives’ realm in both stages.
Marcucci et al.,^38^ Brazil	Adults (18 years or older) living in a community setting, achieving four or more points on the Palliative Care Screening Tool (PCST), being registered in the Estratégia Saúde da Família (ESF) programme, and being willing to complete the Research Consent Form and assessment protocol. The Palliative Care Outcome Scale (POS) was used to assess the key domains in the provision of palliative care (all domains). The Edmonton Symptom Assessment Scale (ESAS; Physical, Psychological) and Karnofsky Performance Scale (KPS; Physical) tools were also used.	Cross- sectional	69 (238)	Community teams were asked to report all patients with a potential need for palliative care, and patients were assessed with structured questionnaires.	Dementia and Cerebrovascular disease were the most frequent conditions of patients identified as needing palliative care. Consequently, most patients had low functional status. Most patients did not have home visits from any health care team member in the month prior to the interview. Assessment of non-physical symptoms like well-being, depression, and anxiety was also limited due to cognitive impairment and the subsequent difficulties in conducting evaluation with the chosen tools. Moreover, no included patients received psychological therapy, and very few had access to multi-professional support.
Mason et al.,^39^ United Kingdom	People with ⩾2 advanced illnesses in a larger multicentre study on care coordination experiences who are considered to be in the last year of life.	Qualitative interviews	37 (56)	Patients and family	Patient experiences of multimorbidity near the end of life were mostly of progressive physical decline with increasingly frequent episodes of acute deterioration, multiple changing medications, progressive loss of autonomy, poor or inconsistent communication, and inadequate continuity of care from numerous but disconnected services. Consequently, the physical and emotional burden of managing care usually fell on the patient or a family carer. Lacking a coherent framework to articulate their experiences and problems, patients and carers tended to interpret their deteriorating health and its consequences as a case of ‘getting old’ rather than being progressively more unwell. As a result, they sometimes failed to seek the right help and support or did not receive it from care systems not configured to respond to their needs. For these people dying from multiple advanced conditions, the daily burdens were great, and crises were common yet there was little evidence of proactive and coordinated services delivered as part of a person-centred palliative care approach.
McVey et al.,^40^ Australia	Residential care home residents aged >18 years classified as needing ‘high-level’ care according to the Residential Classification Scale.	Qualitative interviews	93 (93)	Care home residents and care home staff	The overarching theme from care home resident data was transitioning towards the end of life. Transitioning towards the end of life was seen as a natural progression in life. Embedded within this theme are subthemes: (i) Why am I here?; (ii) Adjusting to life in a mixed-level facility; and (iii) Uncertainty about the future.
Nicholson et al.,^41^ United Kingdom	Patients aged 82–92 years receiving care from a home care service for older people with palliative care needs, living with progressive illness and/or frailty, thought to be in the last year of their life. Participants were included where data was recorded for complete episodes of care between January 2016 and August 2017. The Integrated Palliative Care Outcome Scale (IPOS; all domains), Palliative Care Phase of Illness (POI), and Australia-modified Karnofsky Performance Status (AKPS; Physical) tools were used.	Cross-sectional	815 (2069)	Routinely collected patient data	Standard care patients more frequently reported pain, nausea, vomiting, constipation, anxiety, and family concern and Bromley Care Coordination patients more frequently reported mobility concerns. Older people with multimorbidity, approaching the end of life, have considerable palliative care needs. The expectations were for differences that are more widespread across the two groups since the IPOS tool was developed around the main palliative care concerns of people (including older people) with advanced single diseases.
Österlind et al.,^42^ Sweden	A Nursing Home resident for a maximum period of 6 months before the first interview and fully aware of their orientation in relation to time, space and self, that is without cognitive impairment.	Qualitative interviews	6 (6)	Older people	The narratives revealed that life in a nursing home involved living with a feeling of loneliness in an unfamiliar place. Despite differences in background and health status, the older people described daily life in a nursing home in a similar fashion. They missed their former independence and described how possibilities to affect their everyday lives in the new environment were limited. Daily life was characterised as waiting for death, and as subordinating oneself to the values and norms of the staff while at the same time striving to keep their courage up. They experienced few opportunities to discuss their thoughts on life and death, including preparations for passing away. The study shows that conversation and socialising in everyday life can have an identity-promoting function and promote the feeling of meaningfulness.
Parker et al.,^43^ Australia	Nursing home residents who clinicians would not be surprised if they died within a few months and who had at least one of the following: 1. Multiple admissions to an acute care hospital over the past 6 months, where treatment goals have been palliative not curative; 2. Documented continued trajectory of decline in functional status; 3. Documented recent impaired nutritional status related to progression of the primary disease or other medical condition; 4. An unanticipated medical crisis that required end-of-life care to be discussed. The WARP Karnofsky Performance Score (WKPS; Physical) and Barthel Index (BI; Physical) tools were used.	Qualitative multi-method design (Prospective case notes; resident and staff interviews)	61 (61)	Care home residents and directors of care	Residents who were suffering from multiple conditions were highly dependent for activities of daily living. Their symptoms, in order of prevalence, were constipation, pain, dysphagia, weakness, dyspnoea, and depression. Symptoms such as weakness, anxiety, and restlessness appeared to be the most distressing and the most difficult to relieve. Other symptoms rated as moderate or severe were constipation, dyspnoea, depression, and pain. Residents with moderate/severe symptoms would have been helped by a referral to a respiratory physician (dyspnoea) or to a palliative care service. Most residents’ care was consistent with a palliative approach, with only three residents in the study referred to a specialist palliative care service. However, for some residents, pain and symptom management were not always adequate, and referral to a specialist palliative care service would have been appropriate.
Reinke et al.,^44^ United States of America	Cross-sectional telephone surveys and structured interviews to randomly selected patients at high risk of hospitalisation or death within the subsequent year across four geographical regions of the U.S. enrolled in primary care clinics in the Veterans Affairs Health Care System. The Care Assessment Needs Score (Physical), Veterans Rand 12-item Health Survey (VR12; Physical, Psychological), and Memorial Symptom Assessment Scale (MSAS; Physical and Psychological) tools were used.	Cross-sectional telephone survey	503 (503)	Older people	Patients with multimorbidity (on average five diagnoses) reported high symptom burden, poor physical function, and low quality of life. Among the 503 patients, 26% reported their most bothersome symptom was not assessed by their primary care clinician at their last clinic appointment, and 30% reported this symptom was not being treated. Patients reporting physical symptoms as their most bothersome symptom were more likely to perceive their clinician as addressing their symptoms compared with patients reporting psychological symptoms.
Sloane et al.,^45^ United States of America	Decedents of nursing home residents who had died in the past month, where the resident had spent 15 of the last 30 days of life in a study facility and died no more than 3 days after leaving the facility. The Physician-Family Caregiver Communication (FPPCC; Social, Practical) tool was used.	Cross-sectional	581 (581)	Staff and family (proxy) members of decedents	No differences were noted between decedents with and without dementia in terms of pain, psychosocial status, family involvement in care, advance care planning, use of most life-prolonging interventions, and hospice use. Dying residents with dementia, in comparison with nondemented, tended to die less often in a hospital, have less shortness of breath, receive more physical restraints and sedative medication, and use emergency services less frequently on the last day of life. Persons with dementia dying in residential care/assisted living (RC/AL) tended to have more skin ulcers and poorer hygiene care than non-demented persons in RC/AL.
Strohbuecker et al.,^46^ Germany	Nursing Home residents who suffered from a chronic illness and were able to communicate in a consistent and meaningful way.	Qualitative interviews	9 (9)	Nursing home residents	Six (not mutually exclusive) categories of residents’ needs were identified, encompassing social, emotional, spiritual, and physical aspects of wellbeing. Illness-related issues were not in the foreground; rather, the major concerns were around self-determination of everyday life and social life. Aspects of death and dying were rarely brought up by the participants themselves, but when they were asked explicitly, they were willing to talk about them. Most had a fearless attitude to dying. The residents in this study expressed their needs within their own frame of reference, not using the terminology of medicine, nursing, or palliative care.
van den Brink et al.,^47^ The Netherlands	Nursing home residents who needed both physical and psychiatric care, as shown in the medical history, and whose psychiatric or behavioural problems had been present for ⩾2 years without prospect of substantial recovery. The Camberwell Assessment of Need for the Elderly (CANE; Physical, Practical, Psychological, Social) tool was used.	Mixed methods (convergent design) Survey; patient interviews; staff interviews with clinical notes)	141 (141)	Resident self-report questionnaires and interviews; nursing staff perspectives on residents’ needs through interviews and clinical data review	Discrepancies between residents and nursing staff about perceived unmet needs was most common in the areas of accommodation, company, and daytime activities. Both the residents and the nursing staff reported ‘household activities’, ‘money’, and ‘medication’ as the most frequently met needs. Residents rated the most frequently unmet needs on the domains of ‘accommodation’, ‘company’, and ‘psychological distress’; the nursing staff rated these ‘company’, ‘physical health’, and ‘behaviour’.
Vandenberg et al.,^48^ United States of America	Representatives for deceased military veteran nursing home residents who had died in the preceding year. Representatives were identified by the veterans’ home staff as an individual most attentive to the resident.	Mixed methods (convergent design) Survey; quality improvement (QI); and team activities.	45 (45)	Family or representative of the deceased	The specific areas that were improved per the survey results (which also correlated with staff perceptions) were the following: overall quality of care, spiritual care, distribution of workload, and patients’ preparedness for death. The prevalence of symptoms was reduced by 22% (pain), 25% (dyspnoea), and 30% (uncomfortable symptoms of dying). A marked improvement in involvement of clergy in spiritual care was also noted. The survey process also identified areas that did not improve or worsened, such as management of depression, agitation, anxiety, loneliness, family education, and discussions.
Vohra et al.,^49^ Canada	A family member of a deceased resident of a long-term care facility who had died within the preceding 12 months, had been a resident of the facility for at least a month, and where the death had been expected. Participants were those who were identified by the facility as the family member who possessed power of attorney for personal care, and/or who were viewed by facility staff as most involved in the care of the resident. The Family Perception of Care Scale (FPCS; Physical, Practical) was used.	Survey	104 (213)	A family member of the decedent	Family comments fell into two themes: (1) appreciation for the care and( 2) concerns with care. The appreciation for care theme included the following subthemes: psychosocial support, family care, and spiritual care. The concerns with care theme included the subthemes: physical care, staffing levels, staff knowledge, physician availability, communication, and physical environment.

**Table 4.** Palliative care needs and themes identified.

Domain	Theme	Individual needs	Studies	Total studies
Physical	*Pain*	Pain (including physical discomfort)	Bone et al.^22^, Brandt et al.^23^, Chan and Pang^24^, Chochinov et al.^25^, Kayser-Jones^28^, Kozlov et al.^31^, Kramer^34^, Kricke et al.^35^, Lee et al.^37^, Marcucci et al.^38^, Nicholson et al.^41^, Parker et al.^43^, Sloane et al.^45^, Strohbuecker et al.^46^, Vandenberg et al.^48^, Vohra et al.^49^	17
	*Function*	Activities of daily living	Amblàs-Novellas et al.^20^, Beach et al.^21^, Chan and Pang^24^, Chochinov et al.^25^, Gómez-Batiste et al.^26^, Kendall et al.^30^, Lee et al.^37^, Marcucci et al.^38^, McVey et al.^40^, Österlind et al.^42^, Parker et al.^43^, Sloane et al.^45^, van den Brink et al.^47^	13
		Mobility (including falls)	Amblàs-Novellas et al.^20^, Beach et al.^21^, Chochinov et al.^25^, Gómez-Batiste et al.^26^, Kozlov et al.^31^, Lambotte et al.^36^, Lee et al.^37^, Marcucci et al.^38^, Nicholson et al.^41^, Sloane et al.^45^, Strohbuecker et al.^46^, van den Brink et al.^47^	12
	*Respiratory*	Shortness of breath (including chest infections)	Bone et al.^22^, Brandt et al.^23^, Chochinov et al.^25^, Goodridge et al.^27^, Kozlov et al.^31^, Kramer^34^, Kricke et al.^35^, Lee et al.^37^, Marcucci et al.^38^, Nicholson et al.^41^, Parker et al.^43^, Sloane et al.^45^, Vandenberg et al.^48^, Vohra et al.^49^	14
	*Gastrointestinal*	Nutrition (lack of appetite/anorexia/dysphagia)	Amblàs-Novellas et al.^20^, Brandt et al.^23^, Chan and Pang^24^, Chochinov et al.^25^, Gómez-Batiste et al.^26^, Goodridge et al.^27^, Kayser-Jones^28^, Kayser-Jones et al.^29^, Kramer^34^, Lee et al.^37^, Marcucci et al.^38^, Nicholson et al.^41^, Parker et al.^43^, Sloane et al.^45^	14
		Continence (including urinary tract infections and diarrhoea)	Amblàs-Novellas et al.^20^, Chochinov et al.^25^, Goodridge et al.^27^, Kramer^34^, Lee et al.^37^, Parker et al.^43^, Sloane et al.^45^, van den Brink et al.^47^, Vohra et al.^49^	9
		Constipation	Chochinov et al.^25^, Kayser-Jones^28^, Kramer^34^, Nicholson et al.^41^, Parker et al.^43^	5
		Nausea and vomiting	Chochinov et al.^25^, Parker et al.^43^	2
	*Cognitive*	Dementia, agitation, restlessness, delirium, confusion, and level of consciousness	Amblàs-Novellas et al.^20^, Chochinov et al.^25^, Gómez-Batiste et al.^26^, Kayser-Jones^28^, Kramer^34^, Lee et al.^37^, Marcucci et al.^38^, Parker et al.^43^, Strohbuecker et al.^46^, van den Brink et al.^47^, Vandenberg et al.^48^	11
	*Other physical concerns*	Hygiene (including skin care and oral care)	Amblàs-Novellas et al.^20^, Gómez-Batiste et al.^26^, Goodridge et al.^27^, Kayser-Jones^28^, Kayser-Jones et al.^29^, Kramer^34^, Lee et al.^37^, Parker et al.^43^, Sloane et al.^45^	9
		Lack of energy (including tiredness, fatigue, and trouble sleeping)	Chochinov et al.^25^, Kayser-Jones^28^, Kozlov et al.^31^, Lee et al.^37^, Marcucci et al.^38^, Nicholson et al.^41^, Parker et al.^43^	7
Practical	*Future planning*	Personal affairs (including advance care planning and planning death affairs)	Bone et al.^22^, Brandt et al.^23^, Kendall et al.^30^, Kozlov et al.^31^, Kramer^34^, Lee et al.^37^, Marcucci et al.^38^, McVey et al.^40^, Nicholson et al.^41^, Österlind et al.^42^, Sloane et al.^45^, Strohbuecker et al.^46^, Vandenberg et al.^48^, Vohra et al.^49^, Braun and Clarke^50^	15
		Informational needs	Beach et al.^21^, Bone et al.^22^, Brandt et al.^23^, Kozlov et al.^31^, Lee et al.^37^, Marcucci et al.^38^, Nicholson et al.^41^, Vandenberg et al.^48^	8
		Financial needs	Brandt et al.^23^, Kayser-Jones^28^, Marcucci et al.^38^, Nicholson et al.^41^, Sloane et al.^45^, Strohbuecker et al.^46^, van den Brink et al.^47^	7
		Environmental needs (adaptations/housing/mobility aids/sensory loss)	Beach et al.^21^, Kayser-Jones et al.^29^, Kramer^34^, Lambotte et al.^36^, Mason et al.^39^, Parker et al.^43^, Strohbuecker et al.^46^, van den Brink et al.^47^, Vohra et al.^49^	9
	Person-centred care	Lack of care continuity-across services and/or in-house (including discharge)	Amblàs-Novellas et al.^20^, Bone et al.^22^, Brandt et al.^23^, Goodridge et al.^27^, Kayser-Jones^28^, Kramer^34^, Kricke et al.^35^, Lee et al.^37^, Marcucci et al.^38^, Mason et al.^39^, Strohbuecker et al.^46^	11
		Availability of resources	Beach et al.^21^, Chan and Pang^24^, Kayser-Jones^28^, Kayser-Jones et al.^29^, Kendall et al.^30^, Kozlov et al.^31^, Lee et al.^37^, Marcucci et al.^38^, Vohra et al.^49^	9
		Lack of specialist care/responsive care/professionals skilled in end of life care	Amblàs-Novellas et al.^20^, Bone et al.^22^, Kayser-Jones^28^, Marcucci et al.^38^, Vohra et al.^49^	5
		Medications management	Beach et al.^21^, Kramer^34^, Mason et al.^39^, van den Brink et al.^47^	4
		Wasted time	Brandt et al.^23^, Marcucci et al.^38^	2
Psychological	*Unhappiness*	Emotional distress (including hopelessness)	Amblàs-Novellas et al.^20^, Brandt et al.^23^, Chan and Pang^24^, Chochinov et al.^25^, Gómez-Batiste et al.^26^, Goodridge et al.^27^, Kayser-Jones^28^, Kendall et al.^30^, Kozlov et al.^31^, Marcucci et al.^38^, McVey et al.^40^, Sloane et al.^45^, van den Brink et al.^47^, Vandenberg et al.^48^	14
		General dissatisfaction with life/loss of independence	Chochinov et al.^25^, Kendall et al.^30^, Kozlov et al.^31^, Lambotte et al.^36^, Marcucci et al.^38^, Österlind et al.^42^, Parker et al.^43^, Strohbuecker et al.^46^	8
	*Anxiety*	Anxiety	Brandt et al.^23^, Chochinov et al.^25^, Goodridge et al.^27^, Kayser-Jones^28^, Kozlov et al.^31^, Kramer^34^, Kricke et al.^35^, Lee et al.^37^, Marcucci et al.^38^, Nicholson et al.^41^, Parker et al.^43^, Vandenberg et al.^48^	12
		Anxiety/conflicts related to relationships with family	Brandt et al.^23^, Chochinov et al.^25^, Kozlov et al.^31^, Kramer^34^, Marcucci et al.^38^, Strohbuecker et al.^46^	6
	*Loneliness*	Loneliness/isolation/being ignored	Goodridge et al.^27^, Kayser-Jones^28^, Kayser-Jones et al.^29^, Kendall et al.^30^, Kozlov et al.^31^, Lambotte et al.^36^, Österlind et al.^42^, Parker et al.^43^, Strohbuecker et al.^46^, Vandenberg et al.^48^	10
Social	*Staying socially connected*	Social connection and activity for an elder with family and others (social support/isolation/loneliness)	Beach et al.^21^, Brandt et al.^23^, Chochinov et al.^25^, Kendall et al.^30^, Kozlov et al.^31^, Lee et al.^37^, Marcucci et al.^38^, Nicholson et al.^41^, Österlind et al.^42^, Parker et al.^43^, Sloane et al.^45^, Strohbuecker et al.^46^, van den Brink et al.^47^, Vandenberg et al.^48^	14
		Need for help for an elder on their own/with no carer	Lambotte et al.^36^, Mason et al.^39^	2
	*Maintaining personhood and identity*	Social identity/to be respected	Chochinov et al.^25^, Goodridge et al.^27^, Kendall et al.^30^, Kricke et al.^35^, Österlind et al.^42^, Strohbuecker et al.^46^, van den Brink et al.^47^, Vohra et al.^49^	8
	*Trust in carers*	Need for relational trust and safety with statutory carers (for elder)	Bone et al.^22^, Chochinov et al.^25^, Kayser-Jones^28^, Kramer^34^, Kricke et al.^35^, Lee et al.^37^, Österlind et al.^42^, Strohbuecker et al.^46^	8
		Need for relational trust and safety with statutory carers (for a family member)	Bone et al.^22^, Chochinov et al.^25^, Kayser-Jones^28^, Kramer^34^, Lee et al.^37^, Vohra et al.^49^	6
	*Family support*	Need for the family to be identified as part of the care team and supported	Bone et al.^22^, Kramer^34^, Lambotte et al.^36^, Lee et al.^37^, Mason et al.^39^, Nicholson et al.^41^, Sloane et al.^45^, Vohra et al.^49^	8
		Need for help with family conflict(s) (for elder/between professionals and family)	Kozlov et al.^31^, Lee et al.^37^, Parker et al.^43^, van den Brink et al.^47^	4
Spiritual	*Meaning and purpose*	Need to discuss and/or reconcile the nature of existence (existential distress)	Chan and Pang^24^, Chochinov et al.^25^, Goodridge et al.^27^, Kramer^34^, Lee et al.^37^, Nicholson et al.^41^, Österlind et al.^42^, Sloane et al.^45^, Strohbuecker et al.^46^	9
		Need for meaning and purpose (meaningful activities)	Brandt et al.^23^, Chan and Pang^24^, Chochinov et al.^25^, Kayser-Jones^28^, Kozlov et al.^31^, Lee et al.^37^, Marcucci et al.^38^, Österlind et al.^42^	8
		Need to understand the importance of an individual’s sense of identity (cultural practices and beliefs at end of life)	Chochinov et al.^25^, Kayser-Jones^28^, Kendall et al.^30^, McVey et al.^40^, Österlind et al.^42^, Strohbuecker et al.^46^, Vohra et al.^49^	7
	*Grief and loss*	Need to recognise grief, loss, and deterioration	Chochinov et al.^25^, Kayser-Jones^28^, Kendall et al.^30^, Kramer^34^, Lee et al.^37^, Österlind et al.^42^, Parker et al.^43^, Strohbuecker et al.^46^	8
	*Religiosity and religious preference*	Need to recognise religion and/or religious preferences	Kayser-Jones^28^, Kramer^34^, Kricke et al.^35^, Lee et al.^37^, Parker et al.^43^, Strohbuecker et al.^46^, Vohra et al.^49^	7
	*Living well in the present*	Need for acceptance of living as well as possible in the present	Chochinov et al.^25^, Mason et al.^39^, Strohbuecker et al.^46^	3

## Findings

Figure 1 shows the PRISMA flow diagram for the scoping review that was conducted.

### Study characteristics

Twenty-eight studies were included. Sample size ranged from 6 to 12,481 and presented evidence from 12 countries (Table 3). Study designs included cross-sectional (*N* = 12), qualitative interviews (*N* = 6), mixed or multiple qualitative methods (*N* = 3), mixed (convergent design, qualitative and quantitative) methods (*N* = 3), surveys (*N* = 3) and case control (*N* = 1). Study care settings were described as nursing care homes (*N* = 10), residential care homes (*N* = 5), domiciliary care (*N* = 5), mixed community settings (*N* = 4), and mixed community and acute settings (*N* = 4). Needs were reported by individuals and proxy respondents as follows: older people with multimorbidity (*N* = 7), care professionals (*N* = 6), family members (*N* = 3), older people and family members (*N* = 1), older people and care professionals (*N* = 3), family members and care professionals (*N* = 4), and all groups (*N* = 4).

### Data synthesis

#### Physical needs

Physical needs were reported in all studies. Pain and mobility difficulties were reported most often. Themes for physical needs were ‘pain’, ‘function’, ‘shortness of breath’, ‘gastrointestinal’, and ‘other physical concerns’.

**Pain** was a common experience for older people living with multimorbidity.^43,44,46^

“I have a lot of pain [. . .] I just have to get along with it and make the best of it.”^46^

In one study, pain was found not to be assessed, monitored, and managed adequately for observed care home residents, with 40% experiencing severe pain most of the time.^28^

“Every day the pain just keeps getting worse. They don’t want to give me anything for pain here. I don’t understand. I’m going to die anyway.”^28^

No significant differences in pain reporting between those living with multimorbidity and other groups with life-limiting conditions were found in one study,^25^ while another reported that older people living with multimorbidity receiving palliative care reported pain less frequently compared with standard palliative care patients, although pain was evident in both cohorts.^41^ Pain was frequently cited as the most distressing problem in the final days of life for nursing home residents by family members and nursing home staff.^27^ Another study found uncontrolled pain to contribute to emergency department attendance at the end of life.^22^

**Function** was frequently identified as a need. One study found long-term care home residents with multimorbidity were highly dependent on support to undertake activities of daily living.^43^ While such activities were important to older people living with multimorbidity these were not easily carried out, even when health was stable.^37^ Motor and cognitive impairments often affected the ability to carry out daily activities.^38^ Mobility markedly decreased when patients’ general conditions worsened^37^ and reduced mobility could stem from a range of sources, including joint problems, neurological disorders, weakness, and gait instability.^46^ In one study, older people living with multimorbidity reported mobility concerns more often than standard care palliative patients.^41^ Another study identified limited mobility as an adverse consequence of unmet care needs.^21^

**Respiratory need** with shortness of breath was a common symptom for older veterans with multimorbidity,^44^ and was inadequately treated among people with, and even more so for people without dementia.^45^ People with a primary diagnosis of respiratory disease alongside multimorbidity were more likely to have frequent emergency department attendances.^22^

**Gastrointestinal needs** included nutrition, continence, constipation, nausea, and vomiting. Nutrition was commonly measured. One study recommended educating family members about usual symptoms at the end of life following observation of family distress at reduced oral intake.^27^ Continence and skin integrity were identified as not regularly being assessed or managed.^29^ Nausea and vomiting were sometimes included in multidimensional tools but not reported in detail.

**Cognitive needs** were frequently captured as components within multidimensional measurement tools and in one instance a cognition-specific tool^25^ but rarely in detail to understand the importance of the need for the individual. Proxy responses in one study noted that cognitive impairment was a barrier to the assessment of psychological concerns such as well-being, depression, and anxiety.^38^ Another study reported that many care home residents were cognitively impaired and were often neglected, with any interactions with care professionals made on their behalf often being done by family members.^28^ The same study found that lack of attention to residents’ cognitive status, among other concerns, adversely influenced the experience of dying.^28^

**Other physical concerns** were identified as ‘lack of energy’ and ‘hygiene’. Limited details were identified for these sub-themes as these were mainly items included in multidimensional tools, which received little attention in the papers’ findings. One qualitative study observed that 80% of patients observed in a care home setting appeared to be severely fatigued.^28^ The same study also observed that care to provide adequate oral and skin hygiene had not been given to some care home residents.^28^

#### Practical needs

Practical needs were identified in 25 papers and themes were ‘future planning’ and ‘person-centred care’.

**Future planning** needs were among the most common unmet needs for older adults attending senior centres in one paper.^31^ Some studies found that older people living with multimorbidity did not plan for their deaths,^42^ nor were they offered future care planning,^40^ contributing to anxiety and uncertainty about the future.^40^ Dying was found by family and care professionals to be well managed when older people living with multimorbidity were able to discuss their dying and plan for it.^30^ Financial concerns were commonly perceived needs for care home residents^28,29,47^ and care professionals perceived these as needs that were frequently met.^47^ However, in one study a resident felt that she lacked financial freedom, and that financial restrictions contributed to feelings of shame and reduced control.^46^

**Person-centred care** included multiple elements related to the accessibility and tailored nature of care. Continuity of care across different settings and communication between providers was found to be important for those with multimorbidity to avoid unnecessary hospital readmissions.^22,39^ Having multiple health professionals and poorly coordinated service can lead to mistakes and confusion,^22^ as the following quote illustrates:

“I was given very little notice about his discharge. A doctor told him he could be discharged when he was clearly not well enough to go home.”^22^

Disconnected services can lead to perceptions of inconsistent and impersonal care.^39^ Access to specialist services for long-term care residents was challenging in one study, as services would not provide home visits, and older people living with multimorbidity were unable to travel to access these services.^46^

Inadequate resource and staffing posed challenges to the quality of care received, with adverse outcomes for long-term care residents’ experiences of death,^28,29^ as the following account illustrates:

“During the last week of life, Mrs. Lee’s left arm and hand became edematous; the hospice nurse asked the nursing staff to assist Mrs. Lee with meals, but no one helped her.”^28^

In addition to the above example, care home staff and residents spoke different languages and staff could not respond to residents’ articulated needs. Furthermore, food provision was not culturally appropriate and had an impact on residents’ weight.^28,29^

#### Psychological needs

Psychological needs were identified in 24 papers. Themes were ‘unhappiness’, ‘loneliness’, ‘anxiety’, and ‘depression’.

**Unhappiness** included ‘emotional distress’, ‘upset’, and ‘dissatisfaction’. Unhappiness was reported among the highest unmet care needs by nursing staff^27^ and care home residents,^47^ although a study comparing older people living with frailty with other single disease patient groups found older people did not experience ‘disproportionate psychological distress’.^25^

Having choices and independence was identified as important by older people living with multimorbidity.^43^ Progressive loss of independence caused frustration^30^ and fear,^46^ but dissatisfaction could be tempered by acceptance:

“I’m not really happy here. You just have to accept the situation, after all I get help here . . .”^42^

**Loneliness** was mentioned in some qualitative studies in long-term care settings. Notably within care home environments, some residents felt ‘lonely, alienated and invisible’.^42^ In another study, loneliness and other psychological needs were identified but infrequently assessed or managed by care professionals.^28,29^ Loss of self, through losing personal relations, memories, or possessions were also present for care home residents.^37^

**Anxiety** and **depression** were symptoms commonly rated as moderate or severe in one paper.^43^ A specialist palliative care service found anxiety was evident in all populations, however those with multimorbidity reported anxiety less frequently.^41^ Another study found a positive association between depression and total number of unmet needs, without assigning causality.^47^ Military veterans with greater physical symptom burden often experienced depression.^44^

#### Social needs

Social needs were identified in 22 papers. Themes were ‘staying socially connected’, ‘maintaining personhood and identity’, ‘trust in carers’, and ‘family support’.

**Staying socially connected** was a common unmet care need,^21,25,37^ although the priority for older people living with multimorbidity to stay socially connected was not always recognised as a need by care professionals.^47^ Responsibility for supporting people to maintain social connections was instead frequently discussed in relation to family and friends.^25,43,46^ One paper identified the importance of care professionals’ support in valuing family involvement and making arrangements to include them in care near end of life in a long-term care (LTC) facility, providing support and respite to family members, as shown in this quote:

“The [LTC facility] nurtured not only [the resident] but us as a family. They facilitated all sorts of special arrangements for us so that we could do what [the resident) loved.”^49^

**Maintaining personhood and identity** was difficult to achieve in care homes. One study found care home residents perceived themselves as subordinating themselves to the values and norms of staff.^42^ Another found that care home residents struggled to express themselves and be recognised beyond their illness, both things being important to them.^46^

“There’s very little personal rapport here. There’s nothing personal about the way they talk to you.”^46^

**Trust in carers** Care continuity was found to support the building of trust for older people living with multimorbidity, where trust was identified as the basis for building relationships that foster person-centred care.^46^

“. . .Most of the dying patients come to the point where they don’t trust people; they don’t trust the nurses. And the nurses don’t have much time; there are just an awful lot of patients for the nurses.”^28^

**Family support** was recognised as an important need by older people living with multimorbidity and proxy respondents. Older people and family carers rationalised deteriorating health as part of getting older, with older people often responding by minimising service use and relying more on personal networks and/or family support instead.^39^ Older people living with multimorbidity and their families, living across different care settings, had to deal with ‘growing restrictions on daily activities, loss of family and/or societal roles, increasing dependence, and the emotional demands of a contracting world’.^30^ They responded to this by seeking to stay well and maintain autonomy in the face of increasing dependence.^30^

#### Spiritual needs

Spiritual needs were identified in 19 papers and themes were ‘meaning and purpose (meaningful activities)’, ‘grief and loss’, ‘religiosity and religious preference’, and ‘living well in the present’.

**Meaning and purpose (meaningful activities)** relates to views of self-worth and whether life is worth living.^23,38,39^ This was expressed in various ways including loss of meaning and purpose at the end of life,^31^ reflections about leading a worthwhile life,^38^ the search for more content and meaning in everyday life,^42^ and spiritual pain caused by feelings of meaninglessness,^37^ as the following quote illustrates:

“I’m going to die. Nobody cares for me anymore.”^28^

In contrast, value of life was defined, in one study, as the least desirable quality of life concern for older people living with multimorbidity at end-of-life.^24^ Meaning and purpose were also linked to meaningful activities in life, such as older people being provided with activities they might enjoy,^42^ as the following quote illustrates:

“She wanted to fill her life with more activities and meaning.”^42^

Meaningful activities could also refer to activities that maintain a sense of dignity, such as enabling older people to walk to the toilet rather than insisting on pads^28^ for as long as possible.^39^

**Grief and loss** pertain to the need for older people living with multimorbidity and family members to talk about the impending death and their feelings of grief.^28^ It also relates to older people’s frustrations with declining capacities and losses associated with reduced quality of life,^43^ having less acceptance of dying^30^ and a sense of suffering.^25,37^

**Religiosity and religious preferences** at the end of life were only detailed by one study.^43^ Spiritual needs were mostly recorded as components of multidimensional tools and related to a need to attend religious services and to recognise religious preferences (e.g. last rites).

**Living well in the present** relates to a sense of well-being^25^ or attitude towards the importance of living one’s best possible life in the present moment,^39^ or at least with acceptance of chronic illness^43^ and moderating negative emotion.^24^ One study reports how older people living with frailty, compared with other non-cancer end of life populations, had the highest prevalence of moderate to extreme desire for death and the lowest level of hope.^25^

## Discussion

### Main findings

This paper aims to establish the perspectives of community-dwelling older people living with multimorbidity regarding their palliative care needs. The most identified palliative care needs across domains were pain, function, unhappiness, staying socially connected, future planning, having accessible and tailored care, and having meaning and purpose in life. While palliative care needs were identified across all domains, only 25% of papers drew from research where older people living with multimorbidity themselves solely reported their needs. The remaining 75% of papers reported needs through a proxy (care professionals and/or family), sometimes jointly with older people living with multimorbidity, but most frequently without. Physical needs received the most attention in the reporting of findings; one-third of the tools identified in this review primarily or exclusively reported needs related to physical function. Conversely, expressed physical needs received comparatively less attention in the qualitative accounts of need elicited from older people living with multimorbidity. Explanations for this variance may be (1) physical needs are being met and are therefore no longer of concern, (2) a consequence of non-physical needs being outside the sphere of care professionals’ control, (3) implementation of tools may be driven by the preoccupations of the specific service. Needs reported by older people themselves focussed on psychological symptoms, existential questions, maintaining social connections, being informed about care, and practical issues regarding accessing and receiving timely and trustworthy care tailored to their individual needs and circumstances.

### What this study adds

This is the first paper of which we are aware reporting on the expressed palliative care needs of community-dwelling older people with multimorbidity. The findings highlight the different priorities between the reported items in multidimensional tools used to capture palliative care needs and the needs expressed by older people themselves. Older people living with multimorbidity evidently reported needs that go across domains of need, for example depression, which has a strong correlation with physical function.^51^ Kendall et al.^30^ note the progressive loss of function of people with advanced frailty compared to other advanced disease (cancer and organ failure) and that gradual loss of function, cognitive decline and fear of institutionalisation are more salient needs than needs related to dying. The link between mental and physical health is well established in multimorbidity,^52^ however in this review, cognition is primarily considered as a component of multidimensional tools. There is minimal discussion of the interplay between needs, which illustrates the challenge of whether or not needs are viewed as interconnected.

### Limitations

This scoping review focused only on articles that were published in English. Also, 25 of the studies came from high-income countries; the remaining three were from middle-income countries. A key challenge for this review was to identify relevant papers that focused on the concept of multimorbidity. Historically, multimorbidity has received limited attention in academic literature and has synonymous terms (e.g. co-morbidity) that vary in meaning across different clinical and geographical spaces. Using a published multimorbidity search strategy alongside the scoping review method allowed for development of inclusion/exclusion criteria better to identify the limited evidence available.

Alongside conceptualising multimorbidity, challenges in analysis arose when evidence spanned domains of need, for example the concept of dignity. The flexibility of a scoping review design has enabled development of an analytical framework to encompass diverse data. Further, and crucially for a review focussing on the needs of older people living with multimorbidity from their *own perspectives*, many of the included papers relied on the ‘normative’ needs,^10^ as reported by proxy respondents.

The review also highlights the limits of the available evidence to understand need and thus develop appropriate end of life provision for older people with multimorbidity. Most papers reported using a tool that counted the prevalence of need but gave little detail on the extent or consequences of the need for daily living. Few papers reported directly from older people and more work is required to understand the differences and similarities of needs reported by older people living with multimorbidity themselves and their proxy respondents. Most included papers focus on participants who were living in long-term care settings, with seven of the nine qualitative papers drawing exclusively from long-term care settings. Additional work is also required to understand the differing needs of older people living with multimorbidity in long-term care settings compared to living in other community domiciliary settings.

### Implications for practice

Currently, older people living with multimorbidity are less likely to receive palliative care, and more likely to experience inappropriate, invasive, futile, and costly interventions in their last year of life.^41^ Addressing this inequity includes: (1) new and refined multidimensional tools that reflect and enable identification of what matters most and (2) access to integrated care that spans health and social care provision, which crucially is wrapped around the person in their community to enable them to be an active participant, connected to people and places that are important to them.^53^

Multidimensional patient-reported outcome measures are important in measuring change in health status, quality of life,^54^ and the impact of care. A systematic mixed-methods review of studies evaluating person-centred tools around clinical uncertainty for a similar population highlights the benefits of tools measuring across need domains.^55^ Tools targeting ‘comprehensive assessment and continuity of care’ were found to improve ‘outcomes of quality of death and dying, clinician’s global assessment, goal attainment and symptom burden’.^55^ Any tools developed for older people with multimorbidity near the end of life must cover multiple, complex palliative care needs across domains of need. Further, tools must also measure what matters most to the patients with which they are being used to have validity.^56^ A recent scoping review found that important outcome concepts for older palliative care patients and services were not widely represented within frequently used tools,^57^ drawing attention to the task of correctly measuring the right outcomes. Co-design of tools can support this task, yet in practice this has not been widespread.^58^ A trial of this process with an older population with advancing frailty found that maintaining involvement from older people was essentially dependent on having supportive relatives.^59^ Despite potential barriers, any future tools developed to address the needs of older people with multimorbidity should incorporate co-design. Tools that sit within integrated models of care and incorporate both patient experience and outcomes may be more attuned to capturing and responding to this population’s needs. Older people’s medicine does not, compared with palliative care, have as well-developed tools for capturing multidimensional and patient-focussed needs and outcomes.

Access to end of life care for all people with life limiting conditions is recognised as important at the highest policy level.^60^ However, this remains at odds with structural factors, including over reliance on acute services, dominance of the medical model of care,^61^ ageist practices,^62^ and the persistent view from some that palliative care has connotations with treatment withdrawal and imminent death.^63^ The recent Lancet Commission on revaluing dying^64^ argues that moving beyond the current imbalance and inequity in the experience of dying will require radical shifts including reframing dying as a relational and spiritual process, rather than simply being physiological, and the recognition of an individual’s social network, for example family, friends and communities in leading the support for the dying, rather than professionals. However, radical shifts require an acceptance of mortality, including in older age. Currently, within most discourse there is little, or no, mention of preparation for death as part of healthy ageing.

Access to tailored end of life care for older people requires an interface between geriatric and palliative care and involves core components, including comprehensive assessment, future care planning, and skilled integrated working.^65,66^ Possible changes to clinical practice will vary by locality but can be guided by age-attuned principles of palliative care of relationality, inclusivity, individualisation, and integration, focussed on the needs and concerns of, and delivered in partnership with, older people and their family.^67^ Integrated care for older people living with multimorbidity requires a political, societal, and professional will to invest in the concurrent work of dying and living well in older age.^61^ With increased illness burden, older people living with multimorbidity are disadvantaged in lobbying for equitable care and in services as they are currently configured, which will continue to compound inequity for those living with multimorbidity in older age. Holding together living and dying^68^ requires a recognition of palliative care needs across a complex adaptive system^69,70^ that encompasses dynamic, relational, and social systems, and that integrates and transcends palliative care domains.

## Conclusion

Worldwide, multimorbidity is dramatically increasing, requiring a new and different clinical and service response including at end of life.^71^ The response must begin with older people living with multimorbidity themselves, their understanding of their priorities and palliative care needs. While there is currently limited evidence, this review suggests there are diverging priorities between the dominant ways of identifying and capturing need and the needs that are most important to older people. Refined multidimensional tools and integrated care models are proposed to address unequal palliative care provision for the increasing number of older people living and dying with advancing multimorbidity.

## Supplemental Material

sj-pdf-1-pmj-10.1177_02692163221118230 – Supplemental material for Addressing inequity in palliative care provision for older people living with multimorbidity. Perspectives of community-dwelling older people on their palliative care needs: A scoping review Click here for additional data file. Supplemental material, sj-pdf-1-pmj-10.1177_02692163221118230 for Addressing inequity in palliative care provision for older people living with multimorbidity. Perspectives of community-dwelling older people on their palliative care needs: A scoping review by Caroline Jane Nicholson, Sarah Combes, Freda Mold, Helen King and Richard Green in Palliative Medicine

sj-pdf-2-pmj-10.1177_02692163221118230 – Supplemental material for Addressing inequity in palliative care provision for older people living with multimorbidity. Perspectives of community-dwelling older people on their palliative care needs: A scoping review Click here for additional data file. Supplemental material, sj-pdf-2-pmj-10.1177_02692163221118230 for Addressing inequity in palliative care provision for older people living with multimorbidity. Perspectives of community-dwelling older people on their palliative care needs: A scoping review by Caroline Jane Nicholson, Sarah Combes, Freda Mold, Helen King and Richard Green in Palliative Medicine

sj-pdf-3-pmj-10.1177_02692163221118230 – Supplemental material for Addressing inequity in palliative care provision for older people living with multimorbidity. Perspectives of community-dwelling older people on their palliative care needs: A scoping review Click here for additional data file. Supplemental material, sj-pdf-3-pmj-10.1177_02692163221118230 for Addressing inequity in palliative care provision for older people living with multimorbidity. Perspectives of community-dwelling older people on their palliative care needs: A scoping review by Caroline Jane Nicholson, Sarah Combes, Freda Mold, Helen King and Richard Green in Palliative Medicine
