# Supplementary material for: Addressing inequity in palliative care provision for older people living with multimorbidity. Perspectives of community-dwelling older people on their palliative care needs: A scoping review
Source: Palliat Med. 2022 Aug 24;37(4):475–97. doi: 10.1177/02692163221118230 (PMC10074761; doi:10.1177/02692163221118230)
Supplement: sj-pdf-1-pmj-10.1177_02692163221118230 – Supplemental material for Addressing inequity in palliative care provision for older people living with multimorbidity. Perspectives of community-dwelling older people on their palliative care needs: A scoping review [file sj-pdf-1-pmj-10.1177_02692163221118230.pdf]

Appendix 1: Inclusion and Exclusion Criteria

Category	Inclusion	Exclusion
Older People	More than half the sample is 60 years of age or older. This includes formal and informal caregivers of people aged 60 years of age or older.	Less than half the sample is 60 years of age or older (including formal and informal caregivers of people aged 60 years of age or older);<br>OR<br>The relevant information is not available.
Multi-morbidity[1]	More than half of the sample are older people with two or more chronic conditions, one of which may be author identified frailty, this includes carers of the above;<br>OR<br>Where information on the number and type of conditions is available, multimorbidity is explicitly and relevantly addressed in the analysis and presentation of findings<br>[1]See below	More than half the sample has only one condition;<br>OR<br>No information on number and type of conditions or presence of multimorbidity is available;<br>OR<br>Details are only presented as single disease or where those with multi-morbidity cannot be extracted<br>[1]See below
Palliative and/or End of Life Care	Participants are defined as being in the last 1-2 years of life, or living with advanced disease or frail	Participants are not defined as being at the end of life, or living with advanced disease or frail<br>OR<br>No information is available
Community	The intervention is set in the community, including transitions between community and acute care settings	The study is set in an acute care setting
Study Design	The study presents empirical data	The study is about a specific aspect of end of life care (e.g. delirium, constipation);<br>OR<br>The study does not include empirical evidence (e.g. an editorial)
Needs	The study has a focus on needs and unmet needs from an older person's perspective<br>OR<br>The study has a focus on the needs and unmet needs of older people that is represented by a proxy respondent (e.g. an unpaid carer or care professional)	Needs that are focused on unpaid carers or care professionals rather than the perspective of the older person
Relevance	The study is relevant to the research question.	The study has limited relevance to the research question.

[1]In addition to, or to help the authors to better conceptualise the above, the sampling frame includes the following:

- An answer of 'yes' to the following question supports inclusion (even when the other two multimorbidity inclusion criteria are not met):

- Does this paper help us to understanding the needs of community dwelling older people living with multimorbidity? For instance, studies focussing on single conditions or those related to frailty that have particular relevance to the experiences of older people with multimorbidity (e.g. breathlessness and hospital admission) may warrant inclusion to better understand older people. Relevant topics may include breathlessness, infections, depression, anxiety, unsupported carers, and/or common geriatric symptoms (e.g. frailty, falls, immobility, delirium, incontinence, and susceptibility to side effects of medication).
