# Supplementary material for: Addressing inequity in palliative care provision for older people living with multimorbidity. Perspectives of community-dwelling older people on their palliative care needs: A scoping review
Source: Palliat Med. 2022 Aug 24;37(4):475–97. doi: 10.1177/02692163221118230 (PMC10074761; doi:10.1177/02692163221118230)
Supplement: sj-pdf-2-pmj-10.1177_02692163221118230 – Supplemental material for Addressing inequity in palliative care provision for older people living with multimorbidity. Perspectives of community-dwelling older people on their palliative care needs: A scoping review [file sj-pdf-2-pmj-10.1177_02692163221118230.pdf]

Database(s): Ovid MEDLINE(R) Epub Ahead of Print, In-Process & Other Non-Indexed Citations, Ovid MEDLINE(R) Daily and Ovid MEDLINE(R) 1946 to Present Search Strategy:

#	Searches	Results
1	(comorbid* or co-morbid* or multimorbid* or multi-morbid*).mp. [mp=title, abstract, original title, name of substance word, subject heading word, keyword heading word, protocol supplementary concept word, rare disease supplementary concept word, unique identifier, synonyms]	197654
2	(multidisease* or multi-disease* or (multiple adj3 (illness* or disease* or condition* or syndrom* or disorder*))).mp. [mp=title, abstract, original title, name of substance word, subject heading word, keyword heading word, protocol supplementary concept word, rare disease supplementary concept word, unique identifier, synonyms]	41895
3	exp Comorbidity/	91638
4	1 or 2 or 3	236961
5	((chronic* or long-term) adj3 (disease* or condition* or disorder* or ill*)).mp. [mp=title, abstract, original title, name of substance word, subject heading word, keyword heading word, protocol supplementary concept word, rare disease supplementary concept word, unique identifier, synonyms]	511713
6	exp Chronic Disease/	246536
7	5 or 6	511776
8	(Non-malignan* or non-cancer* or non-oncolog*).mp. [mp=title, abstract, original title, name of substance word, subject heading word, keyword heading word, protocol supplementary concept word, rare disease supplementary concept word, unique identifier, synonyms]	16470
9	4 or 7 or 8	736905
10	exp Diabetes Mellitus/ or diabet*.mp.	602303
11	(hypertens* or high blood pressure).mp. [mp=title, abstract, original title, name of substance word, subject heading word, keyword heading word, protocol supplementary concept word, rare disease supplementary concept word, unique identifier, synonyms]	464428
12	exp Hypertension/	237496
13	11 or 12	464428
14	((heart* or cardiac or cardiovascular or coronary) adj3 (disease* or disorder* or failure* or insufficien* or arrhythmia*)).mp. [mp=title, abstract, original title, name of substance word, subject heading word, keyword heading word, protocol supplementary concept word, rare disease supplementary concept word, unique identifier, synonyms]	816938
15	exp Heart Diseases/	1028300
16	14 or 15	1327275
17	((kidney or renal) adj3 (disease* or disorder* or failure* or insufficien*)).mp. [mp=title, abstract, original title, name of substance word, subject heading word, keyword heading word, protocol supplementary concept word, rare disease supplementary concept word, unique identifier, synonyms]	319721
18	exp Kidney Diseases/	470411
19	17 or 18	546018
20	((liver or hepatic) adj3 (disease* or disorder* or failure* or insufficien*)).mp. [mp=title, abstract, original title, name of substance word, subject heading word, keyword heading word, protocol supplementary concept word, rare disease supplementary concept word, unique identifier, synonyms]	172638
21	exp Liver Diseases/	503826
22	20 or 21	542246
23	(((chronic* or obstruct*) adj3 (pulmonary or lung* or airway* or bronch* or respirator*)) or COPD).mp. [mp=title, abstract, original title, name of substance word, subject heading word, keyword heading word, protocol supplementary concept word, rare disease supplementary concept word, unique identifier, synonyms]	142137
24	exp Pulmonary Disease, Chronic Obstructive/	47457
25	emphysema*.mp. or exp Pulmonary Emphysema/	33328
26	asthma*.mp. or exp Asthma/	162996
27	23 or 24 or 25 or 26	306932
28	(stroke or ((cerebrovascular or brain) adj3 (isch?emia* or h?emorrhage* or infarction*))).mp. [mp=title, abstract, original title, name of substance word, subject heading word, keyword heading word, protocol supplementary concept word, rare disease supplementary concept word, unique identifier, synonyms]	280626
29	((cerebrovascular or vascular or carotid* or arter*) adj3 (disease* or disorder*)).mp. [mp=title, abstract, original title, name of substance word, subject heading word, keyword heading word, protocol supplementary concept word, rare disease supplementary concept word, unique identifier, synonyms]	309102
30	exp Cerebrovascular Disorders/	327427
31	28 or 29 or 30	668270
32	exp Arthritis, Rheumatoid/ or arthritis.mp.	211601
33	exp Osteoporosis/ or osteoporosis.mp.	76769
34	exp Musculoskeletal Diseases/ or muscoloskeletal.mp.	992313
35	32 or 33 or 34	1052644
36	depression.mp. or exp Depression/	347773
37	(dementia or Alzheimer* or senil*).mp. [mp=title, abstract, original title, name of substance word, subject heading word, keyword heading word, protocol supplementary concept word, rare disease supplementary concept word, unique identifier, synonyms]	210345
38	exp Dementia/ or exp Alzheimer Disease/	144226
39	37 or 38	226417

40	Parkinson*.mp. or exp Parkinson Disease/	110483
41	(frail* or frailty syndrome).mp. [mp=title, abstract, original title, name of substance word, subject heading word, keyword heading word, protocol supplementary concept word, rare disease supplementary concept word, unique identifier, synonyms]	19545
42	10 or 13 or 16 or 19 or 22 or 27 or 31 or 35 or 36 or 39 or 40 or 41	5128666
43	((co-occur* or cooccur* or co-exist* or coexist* or multipl*) adj3 (disease* or condition* or disorder* or ill* or symptom* or medication* or care*)).mp. [mp=title, abstract, original title, name of substance word, subject heading word, keyword heading word, protocol supplementary concept word, rare disease supplementary concept word, unique identifier, synonyms]	57139
44	(chronic or long-term).mp. [mp=title, abstract, original title, name of substance word, subject heading word, keyword heading word, protocol supplementary concept word, rare disease supplementary concept word, unique identifier, synonyms]	1795626
45	43 or 44	1841331
46	42 and 45	811694
47	(Old* person* or old* people or elder* or old* adult*).mp. [mp=title, abstract, original title, name of substance word, subject heading word, keyword heading word, protocol supplementary concept word, rare disease supplementary concept word, unique identifier, synonyms]	308705
48	exp Aged/	2775459
49	47 or 48	2860899
50	(Palliative care or support* care or terminal care or end-of-life or end of life or EOL or terminal* ill* or death or dying).mp. [mp=title, abstract, original title, name of substance word, subject heading word, keyword heading word, protocol supplementary concept word, rare disease supplementary concept word, unique identifier, synonyms]	782868
51	exp Palliative Care/	47701
52	exp Terminal Care/	46654
53	50 or 51 or 52	791036
54	(Home-based or homebased).mp. [mp=title, abstract, original title, name of substance word, subject heading word, keyword heading word, protocol supplementary concept word, rare disease supplementary concept word, unique identifier, synonyms]	8025
55	exp Home Care Services/	44031
56	54 or 55	49781
57	((Primary adj3 care) or general practic* or generalist* or GP* or family practic* or family physician*).mp. [mp=title, abstract, original title, name of substance word, subject heading word, keyword heading word, protocol supplementary concept word, rare disease supplementary concept word, unique identifier, synonyms]	388165
58	((Community adj3 service*) or community-based or community-dwell* or community nurs* or community health nurs* or community health vis* or district nurse*).mp. [mp=title, abstract, original title, name of substance word, subject heading word, keyword heading word, protocol supplementary concept word, rare disease supplementary concept word, unique identifier, synonyms]	140308
59	exp Community Health Services/	276825
60	57 or 58 or 59	703871
61	(Care home* or nursing home* or residential care home* or sheltered hous*).mp. [mp=title, abstract, original title, name of substance word, subject heading word, keyword heading word, protocol supplementary concept word, rare disease supplementary concept word, unique identifier, synonyms]	44457
62	exp Nursing Homes/	36058
63	exp Homes for the Aged/	12713
64	exp Residential Facilities/	48386
65	61 or 62 or 63 or 64	59596
66	exp Social Work/ or social work*.mp.	24704
67	volunteer*.mp. or exp Volunteers/	186581
68	56 or 60 or 65 or 66 or 67	954537
69	exp MULTIMORBIDITY/	51
70	9 or 69	736905
71	49 and 53 and 68 and 70	2276
72	46 and 49 and 53 and 68	1797
73	71 or 72	3026
74	limit 73 to english language	2832
75	exp Needs Assessment/	26847
76	needs assessment.mp. [mp=title, abstract, original title, name of substance word, subject heading word, keyword heading word, protocol supplementary concept word, rare disease supplementary concept word, unique identifier, synonyms]	30080
77	needs.mp. [mp=title, abstract, original title, name of substance word, subject heading word, keyword heading word, protocol supplementary concept word, rare disease supplementary concept word, unique identifier, synonyms]	322067
78	desire.mp. [mp=title, abstract, original title, name of substance word, subject heading word, keyword heading word, protocol supplementary concept word, rare disease supplementary concept word, unique identifier, synonyms]	27890
79	preferences.mp. [mp=title, abstract, original title, name of substance word, subject heading word, keyword heading word, protocol supplementary concept word, rare disease supplementary concept word, unique identifier, synonyms]	62147

80	wants.mp. [mp=title, abstract, original title, name of substance word, subject heading word, keyword heading word, protocol supplementary concept word, rare disease supplementary concept word, unique identifier, synonyms]	4045
81	patient needs.mp. [mp=title, abstract, original title, name of substance word, subject heading word, keyword heading word, protocol supplementary concept word, rare disease supplementary concept word, unique identifier, synonyms]	2968
82	exp "Health Services Needs and Demand"/	55602
83	health services need.mp. [mp=title, abstract, original title, name of substance word, subject heading word, keyword heading word, protocol supplementary concept word, rare disease supplementary concept word, unique identifier, synonyms]	165
84	75 or 76 or 77 or 78 or 79 or 80 or 81 or 82 or 83	412254
85	74 and 84	413

Result 1. Unique Identifier

29471360

Authors Beach SR; Schulz R; Friedman EM; Rodakowski J; Martsolf G; James AE 3rd.

Authors Full Name Beach, Scott R; Schulz, Richard; Friedman, Esther M; Rodakowski, Juleen; Martsolf, Grant; James, Alton Everette 3rd.

Title Adverse Consequences of Unmet Needs for Care in High-Need/High-Cost Older Adults.

Source Journals of Gerontology Series B-Psychological Sciences & Social Sciences. 2018 Feb 17.

Publication Type Journal Article.

Result 2.

Unique Identifier 29336103

Authors Chan HY; Chun GK; Man CW; Leung EM.

Authors Full Name Chan, Helen Yl; Chun, Gloria Km; Man, C W; Leung, Edward Mf.

Title Staff preparedness for providing palliative and end-of-life care in long-term care homes: Instrument development and validation.

Source Geriatrics & gerontology international. 2018 Jan 16.

Publication Type Journal Article.

Result 3.

Unique Identifier 29140508

Authors Sable-Smith A; Arnett KR; Nowels MA; Colborn K; Lum HD; Nowels D.

Authors Full Name Sable-Smith, Alex; Arnett, Kelly R; Nowels, Molly A; Colborn, Kathryn; Lum, Hillary D; Nowels, David.

Title Interactions with the healthcare system influence advance care planning activities: results from a representative survey in 11 developed countries.

Source Family Practice. 2017 Nov 13.

Publication Type Journal Article.

Result 4.

Unique Identifier 29206578

Authors Maldonado LY; Goodson RB; Mulroy MC; Johnson EM; Reilly JM; Homeier DC.

Authors Full Name Maldonado, Lauren Y; Goodson, Ruth B; Mulroy, Matthew C; Johnson, Emily M; Reilly, Jo M; Homeier, Diana C.

Title Wellness in Sickness and Health (The W.I.S.H. Project): Advance Care Planning Preferences and Experiences Among Elderly Latino Patients.

Source Clinical Gerontologist. 1-8, 2017 Oct 25.

Publication Type Journal Article.

Result 5.

Unique Identifier 29151044

Authors Lakkappa B; Shah S; Rogers S; Holman LH.

Authors Full Name Lakkappa, Bharath; Shah, Sanjay; Rogers, Stephen; Holman, Leanne Helen.

Title Mortality among referrals to a community-based intermediate care team.

Source BMJ supportive & palliative care. 2017 Nov 18.

Publication Type Journal Article.

Result 6.

Unique Identifier	27402261
Authors	Afshar K; Geiger K; Muller-Mundt G; Bleidorn J; Schneider N.
Authors Full Name	Afshar, K; Geiger, K; Muller-Mundt, G; Bleidorn, J; Schneider, N.
Title	Generalist palliative care for non-cancer patients : A review article.
Source	Der Schmerz. 2016 Jul 11.
Publication Type	Journal Article.

Result 7.

Unique Identifier	26247082
Authors	Pollock K; Wilson E.
Authors Full Name	Pollock, Kristian; Wilson, Eleanor.
Book Title	Care and communication between health professionals and patients affected by severe or chronic illness in community care settings: a qualitative study of care at the end of life
Source	NIHR Journals Library. Health Services and Delivery Research 2015 07.
Publication Type	Review.

Result 8.

Unique Identifier	25642566
Authors	Hanratty B; Lowson E; Grande G; Payne S; Addington-Hall J; Valtorta N; Seymour J.
Authors Full Name	Hanratty, Barbara; Lowson, Elizabeth; Grande, Gunn; Payne, Sheila; Addington-Hall, Julia; Valtorta, Nicole; Seymour, Jane.
Book Title	Transitions at the end of life for older adults - patient, carer and professional perspectives: a mixed-methods study
Source	NIHR Journals Library. Health Services and Delivery Research 2014 06.
Publication Type	Review.

Result 9.

Unique Identifier	27606397
Authors	Peterson K; Helfand M; Humphrey L; Christensen V; Carson S.
Authors Full Name	Peterson, Kim; Helfand, Mark; Humphrey, Linda; Christensen, Vivian; Carson, Susan.
Title	Evidence Brief: Effectiveness of Intensive Primary Care Programs. [Review]
Book Title	VA Evidence-based Synthesis Program Evidence Briefs
Source	Department of Veterans Affairs (US). VA Evidence-based Synthesis Program Reports 2011.
Publication Type	Review.

Result 10.

Unique Identifier	28876730
Authors	Swedish Council on Health Technology Assessment.
Authors Full Name	Swedish Council on Health Technology Assessment.
Book Title	Peripheral Arterial Disease - Diagnosis and Treatment: A Systematic Review
Source	Swedish Council on Health Technology Assessment (SBU). SBU Systematic Review Summaries, SBU Yellow Report No. 187. 2008 11.
Publication Type	Review.

Result 11.

Unique Identifier	27496356
Authors	Chapman M; Johnston N; Lovell C; Forbat L; Liu WM.
Authors Full Name	Chapman, Michael; Johnston, Nikki; Lovell, Clare; Forbat, Liz; Liu, Wai-Man.
Title	Avoiding costly hospitalisation at end of life: findings from a specialist palliative care pilot in residential care for older adults.
Source	BMJ supportive & palliative care. 8(1):102-109, 2018 Mar.
Publication Type	Journal Article.

Result 12.

Unique Identifier	28960161
Authors	Adogwa O; Elsamadicy AA; Vuong VD; Moreno J; Cheng J; Karikari IO; Bagley CA.
Authors Full Name	Adogwa, Owoicho; Elsamadicy, Aladine A; Vuong, Victoria D; Moreno, Jessica; Cheng, Joseph; Karikari, Isaac O; Bagley, Carlos A.
Title	Geriatric comanagement reduces perioperative complications and shortens duration of hospital stay after lumbar spine surgery: a prospective single-institution experience.
Source	Journal of Neurosurgery Spine. 27(6):670-675, 2017 Dec.
Publication Type	Journal Article.

Result 13.

Unique Identifier	29179710
Authors	Payne S; Eastham R; Hughes S; Varey S; Hasselaar J; Preston N.
Authors Full Name	Payne, Sheila; Eastham, Rachael; Hughes, Sean; Varey, Sandra; Hasselaar, Jeroen; Preston, Nancy.
Title	Enhancing integrated palliative care: what models are appropriate? A cross-case analysis.
Source	BMC Palliative Care. 16(1):64, 2017 Nov 28.
Publication Type	Journal Article.

Result 14.

Unique Identifier	29169346
Authors	Higginson IJ; Daveson BA; Morrison RS; Yi D; Meier D; Smith M; Ryan K; McQuillan R; Johnston BM; Normand C; BuildCARE.
Authors Full Name	Higginson, Irene J; Daveson, Barbara A; Morrison, R Sean; Yi, Deokhee; Meier, Diane; Smith, Melinda; Ryan, Karen; McQuillan, Regina; Johnston, Bridget M; Normand, Charles; BuildCARE.
Title	Social and clinical determinants of preferences and their achievement at the end of life: prospective cohort study of older adults receiving palliative care in three countries.
Source	BMC Geriatrics. 17(1):271, 2017 Nov 23.
Publication Type	Journal Article.

Result 15.

Unique Identifier	29061178
Authors	Gonzalez K; Ulloa JG; Moreno G; Echeverria O; Norris K; Talamantes E.
Authors Full Name	Gonzalez, Karla; Ulloa, Jesus G; Moreno, Gerardo; Echeverria, Oscar; Norris, Keith; Talamantes, Efrain.
Title	Intensive procedure preferences at the end of life (EOL) in older Latino adults with end stage renal disease (ESRD) on dialysis.
Source	BMC Nephrology. 18(1):319, 2017 Oct 23.
Publication Type	Journal Article.

Result 16.

Unique Identifier	28933993
Authors	Finucane AM; Stevenson B; Murray SA.
Authors Full Name	Finucane, Anne M; Stevenson, Barbara; Murray, Scott A.
Title	Gradual physical decline characterises the illness trajectories of care home residents.
Source	International Journal of Palliative Nursing. 23(9):457-461, 2017 Sep 02.
Publication Type	Journal Article.

Result 17.

Unique Identifier	28209982
Authors	Chen YC; Weng SC; Liu JS; Chuang HL; Hsu CC; Tarng DC.
Authors Full Name	Chen, Yi-Chi; Weng, Shuo-Chun; Liu, Jia-Sin; Chuang, Han-Lin; Hsu, Chih-Cheng; Tarng, Der-Cherng.
Title	Severe Decline of Estimated Glomerular Filtration Rate Associates with Progressive Cognitive Deterioration in the Elderly: A Community-Based Cohort Study.
Source	Scientific Reports. 7:42690, 2017 Feb 17.
Publication Type	Journal Article.

Result 18.

Unique Identifier	24188214
Authors	Abba K; Byrne P; Horton S; Lloyd-Williams M.
Authors Full Name	Abba, Katharine; Byrne, Paula; Horton, Siobhan; Lloyd-Williams, Mari.
Title	Interventions to encourage discussion of end-of-life preferences between members of the general population and the people closest to them - a systematic literature review.
Source	BMC Palliative Care. 12(1):40, 2013 Nov 04.
Publication Type	Journal Article.

Result 19.

Unique Identifier	24653231
Authors	Pinnock H; Kendall M; Murray SA; Worth A; Levack P; Porter M; MacNee W; Sheikh A.
Authors Full Name	Pinnock, Hilary; Kendall, Marilyn; Murray, Scott A; Worth, Allison; Levack, Pamela; Porter, Mike; MacNee, William; Sheikh, Aziz.
Title	Living and dying with severe chronic obstructive pulmonary disease: multi-perspective longitudinal qualitative study. [Reprint of BMJ. 2011;342:d142; PMID: 21262897]
Source	BMJ supportive & palliative care. 1(2):174-83, 2011 Sep.
Publication Type	Journal Article.

Result 20.

Unique Identifier	21827696
Authors	Duursma F; Schers HJ; Vissers KC; Hasselaar J.
Authors Full Name	Duursma, Froukje; Schers, Henk J; Vissers, Kris Cp; Hasselaar, Jeroen.
Title	Study protocol: optimization of complex palliative care at home via telemedicine. A cluster randomized controlled trial.
Source	BMC Palliative Care. 10:13, 2011 Aug 09.
Publication Type	Journal Article.

Result 21.

Unique Identifier	21388538
Authors	Gysels M; Pell C; Straus L; Pool R.
Authors Full Name	Gysels, Marjolein; Pell, Christopher; Straus, Lianne; Pool, Robert.
Title	End of life care in sub-Saharan Africa: a systematic review of the qualitative literature.
Source	BMC Palliative Care. 10:6, 2011 Mar 09.
Publication Type	Journal Article.

Result 22.

Unique Identifier	23074509
Authors	Health Quality Ontario.
Authors Full Name	Health Quality Ontario.
Title	Caregiver- and patient-directed interventions for dementia: an evidence-based analysis.
Source	Ontario Health Technology Assessment Series. 8(4):1-98, 2008.
Publication Type	Journal Article.

Result 23.

Unique Identifier	28056634
Authors	van der Plas AG; Oosterveld-Vlug MG; Pasman HR; Onwuteaka-Philipsen BD.
Authors Full Name	van der Plas, Annicka Gm; Oosterveld-Vlug, Mariska G; Pasman, H Roeline W; Onwuteaka-Philipsen, Bregje D.
Title	Relating cause of death with place of care and healthcare costs in the last year of life for patients who died from cancer, chronic obstructive pulmonary disease, heart failure and dementia: A descriptive study using registry data.
Source	Palliative Medicine. 31(4):338-345, 2017 Apr.
Publication Type	Comparative Study. Journal Article.

Result 24.

Unique Identifier	27495813
Authors	Lovell N; Jones C; Baynes D; Dinning S; Vinen K; Murtagh FE.
Authors Full Name	Lovell, Natasha; Jones, Chris; Baynes, Dawn; Dinning, Sarah; Vinen, Katie; Murtagh, Fliss Em.
Title	Understanding patterns and factors associated with place of death in patients with end-stage kidney disease: A retrospective cohort study. [Review]
Source	Palliative Medicine. 31(3):283-288, 2017 Mar.
Publication Type	Journal Article. Review.

Result 25.

Unique Identifier	27885156
Authors	McNiel P; Westphal J.
Authors Full Name	McNiel, Paula; Westphal, Judith.
Title	Namaste CareTM: A Person-Centered Care Approach for Alzheimer's and Advanced Dementia.
Source	Western Journal of Nursing Research. 40(1):37-51, 2018 Jan.
Publication Type	Journal Article.

Result 26.

Unique Identifier	28279992
Authors	Browne J; Edwards DA; Rhodes KM; Brimicombe DJ; Payne RA.
Authors Full Name	Browne, Jorge; Edwards, Duncan A; Rhodes, Kirsty M; Brimicombe, D James; Payne, Rupert A.
Title	Association of comorbidity and health service usage among patients with dementia in the UK: a population-based study.
Source	BMJ Open. 7(3):e012546, 2017 03 09.
Publication Type	Journal Article. Multicenter Study.

Result 27.

Unique Identifier	26657464
Authors	Nguyen M; Pachana NA; Beattie E; Fielding E; Ramis MA.
Authors Full Name	Nguyen, Mynhi; Pachana, Nancy A; Beattie, Elizabeth; Fielding, Elaine; Ramis, Mary-Anne.
Title	Effectiveness of interventions to improve family-staff relationships in the care of people with dementia in residential aged care: a systematic review protocol. [Review]
Source	JBI Database Of Systematic Reviews And Implementation Reports. 13(11):52-63, 2015 Nov.
Publication Type	Journal Article. Review.

Result 28.

Unique Identifier	27834616
Authors	Inaba M.
Authors Full Name	Inaba, Miyuki.
Title	Aging and Elder Care in Japan: A Call for Empowerment-Oriented Community Development.
Source	Journal of Gerontological Social Work. 59(7-8):587-603, 2016 Oct - Nov.
Publication Type	Journal Article.

Result 29.

Unique Identifier	27645556
Authors	Amblas-Novellas J; Murray SA; Espaulella J; Martori JC; Oller R; Martinez-Munoz M; Molist N; Blay C; Gomez-Batiste X.
Authors Full Name	Amblas-Novellas, J; Murray, S A; Espaulella, J; Martori, J C; Oller, R; Martinez-Munoz, M; Molist, N; Blay, C; Gomez-Batiste, X.
Title	Identifying patients with advanced chronic conditions for a progressive palliative care approach: a cross-sectional study of prognostic indicators related to end-of-life trajectories.
Source	BMJ Open. 6(9):e012340, 2016 09 19.
Publication Type	Journal Article. Research Support, Non-U.S. Gov't.

Result 30.

Unique Identifier	27064659
Authors	Morisset J; Dube BP; Garvey C; Bourbeau J; Collard HR; Swigris JJ; Lee JS.
Authors Full Name	Morisset, Julie; Dube, Bruno-Pierre; Garvey, Chris; Bourbeau, Jean; Collard, Harold R; Swigris, Jeffrey J; Lee, Joyce S.
Title	The Unmet Educational Needs of Patients with Interstitial Lung Disease. Setting the Stage for Tailored Pulmonary Rehabilitation.
Source	Annals of the American Thoracic Society. 13(7):1026-33, 2016 Jul.
Publication Type	Journal Article. Multicenter Study.

Result 31.

Unique Identifier	28991827
Authors	Ehlenbach WJ; Gilmore-Bykovskyi A; Repplinger MD; Westergaard RP; Jacobs EA; Kind AJH; Smith M.
Authors Full Name	Ehlenbach, William J; Gilmore-Bykovskyi, Andrea; Repplinger, Michael D; Westergaard, Ryan P; Jacobs, Elizabeth A; Kind, Amy J H; Smith, Maureen.
Title	Sepsis Survivors Admitted to Skilled Nursing Facilities: Cognitive Impairment, Activities of Daily Living Dependence, and Survival.
Source	Critical Care Medicine. 46(1):37-44, 2018 Jan.
Publication Type	Comparative Study. Journal Article.

Result 32.

Unique Identifier	28145084
Authors	Valery PC; Clark PJ; McPhail SM; Rahman T; Hayward K; Martin J; Horsfall L; Volk ML; Skoien R; Powell E.
Authors Full Name	Valery, Patricia C; Clark, Paul J; McPhail, Steven M; Rahman, Tony; Hayward, Kelly; Martin, Jennifer; Horsfall, Leigh; Volk, Michael L; Skoien, Richard; Powell, Elizabeth.
Title	Exploratory study into the unmet supportive needs of people diagnosed with cirrhosis in Queensland, Australia.
Source	Internal Medicine Journal. 47(4):429-435, 2017 Apr.
Publication Type	Journal Article.

Result 33.

Unique Identifier	29229128
Authors	Fernandes-Taylor S; Berg S; Gunter R; Bennett K; Smith MA; Rathouz PJ; Greenberg CC; Kent KC.
Authors Full Name	Fernandes-Taylor, Sara; Berg, Stephen; Gunter, Rebecca; Bennett, Kyla; Smith, Maureen A; Rathouz, Paul J; Greenberg, Caprice C; Kent, K Craig.
Title	Thirty-day readmission and mortality among Medicare beneficiaries discharged to skilled nursing facilities after vascular surgery.
Source	Journal of Surgical Research. 221:196-203, 2018 Jan.
Publication Type	Journal Article.

Result 34.

Unique Identifier	28495366
Authors	Slomka J; Prince-Paul M; Webel A; Daly BJ.
Authors Full Name	Slomka, Jacquelyn; Prince-Paul, Maryjo; Webel, Allison; Daly, Barbara J.
Title	Multimorbidity With HIV: Views of Community-Based People Living With HIV and Other Chronic Conditions.
Source	Journal of the Association of Nurses in AIDS Care. 28(4):603-611, 2017 Jul - Aug.
Publication Type	Journal Article.

Result 35.

Unique Identifier	28991827
Authors	Ehlenbach WJ; Gilmore-Bykovskyi A; Repplinger MD; Westergaard RP; Jacobs EA; Kind AJH; Smith M.
Authors Full Name	Ehlenbach, William J; Gilmore-Bykovskyi, Andrea; Repplinger, Michael D; Westergaard, Ryan P; Jacobs, Elizabeth A; Kind, Amy J H; Smith, Maureen.
Title	Sepsis Survivors Admitted to Skilled Nursing Facilities: Cognitive Impairment, Activities of Daily Living Dependence, and Survival.
Source	Critical Care Medicine. 46(1):37-44, 2018 Jan.
Publication Type	Comparative Study. Journal Article.

Result 36.

Unique Identifier	26808530
Authors	Chochinov HM; Johnston W; McClement SE; Hack TF; Dufault B; Enns M; Thompson G; Harlos M; Damant RW; Ramsey CD; Davison S; Zacharias J; Milke D; Strang D; Campbell-Enns HJ; Kredentser MS.
Authors Full Name	Chochinov, Harvey Max; Johnston, Wendy; McClement, Susan E; Hack, Thomas F; Dufault, Brenden; Enns, Murray; Thompson, Genevieve; Harlos, Mike; Damant, Ronald W; Ramsey, Clare D; Davison, Sara; Zacharias, James; Milke, Doris; Strang, David; Campbell-Enns, Heather J; Kredentser, Maia S.
Title	Dignity and Distress towards the End of Life across Four Non-Cancer Populations.[Erratum appears in PLoS One. 2017 Nov 9;12 (11):e0188141; PMID: 29121648]
Source	PLoS ONE [Electronic Resource]. 11(1):e0147607, 2016.
Publication Type	Journal Article. Multicenter Study. Research Support, Non-U.S. Gov't.

Result 37.

Unique Identifier	28295138
Authors	Russell D; Diamond EL; Lauder B; Dignam RR; Dowding DW; Peng TR; Prigerson HG; Bowles KH.
Authors Full Name	Russell, David; Diamond, Eli L; Lauder, Bonnie; Dignam, Ritchell R; Dowding, Dawn W; Peng, Timothy R; Prigerson, Holly G; Bowles, Kathryn H.
Title	Frequency and Risk Factors for Live Discharge from Hospice.
Source	Journal of the American Geriatrics Society. 65(8):1726-1732, 2017 Aug.
Publication Type	Journal Article.

Result 38.

Unique Identifier	27932338
Authors	Marx G; Nasse M; Stanze H; Boakye SO; Nauck F; Schneider N.
Authors Full Name	Marx, Gabriella; Nasse, Maximilian; Stanze, Henrikje; Boakye, Sonja Owusu; Nauck, Friedemann; Schneider, Nils.
Title	Meaning of living with severe chronic obstructive lung disease: a qualitative study.
Source	BMJ Open. 6(12):e011555, 2016 12 08.
Publication Type	Journal Article. Research Support, Non-U.S. Gov't.

Result 39.

Unique Identifier	19417860
Authors	Campbell NR; Leiter LA; Larochelle P; Tobe S; Chockalingam A; Ward R; Morris D; Tsuyuki R.
Authors Full Name	Campbell, Norman R C; Leiter, Lawrence A; Larochelle, Pierre; Tobe, Sheldon; Chockalingam, Arun; Ward, Richard; Morris, Dorothy; Tsuyuki, Ross.
Title	Hypertension in diabetes: a call to action. [Review] [48 refs]
Source	Canadian Journal of Cardiology. 25(5):299-302, 2009 May.
Publication Type	Journal Article. Review.

Result 40.

Unique Identifier	17932591
Authors	Champagne J; Philippon F; Gilbert M; Molin F; Blier L; Nault I; Sarrazin JF; Charbonneau L; Dufort L; Drolet B; Chahine M; O'Hara GE.
Authors Full Name	Champagne, Jean; Philippon, Francois; Gilbert, Marcel; Molin, Franck; Blier, Louis; Nault, Isabelle; Sarrazin, Jean-Francois; Charbonneau, Lyne; Dufort, Line; Drolet, Benoit; Chahine, Mohamed; O'Hara, Gilles E.
Title	The Brugada syndrome in Canada: a unique French-Canadian experience.[Erratum appears in Can J Cardiol. 2009 Mar;25(3):140]
Source	Canadian Journal of Cardiology. 23 Suppl B:71B-75B, 2007 Oct.
Publication Type	Journal Article. Research Support, Non-U.S. Gov't.

Result 41.

Unique Identifier	28892128
Authors	Emerson JA; Smith CY; Long KH; Ransom JE; Roberts RO; Hass SL; Duhig AM; Petersen RC; Leibson CL.
Authors Full Name	Emerson, Jane A; Smith, Carin Y; Long, Kirsten H; Ransom, Jeanine E; Roberts, Rosebud O; Hass, Steven L; Duhig, Amy M; Petersen, Ronald C; Leibson, Cynthia L.

Title	Nursing Home Use Across The Spectrum of Cognitive Decline: Merging Mayo Clinic Study of Aging With CMS MDS Assessments.
Source	Journal of the American Geriatrics Society. 65(10):2235-2243, 2017 Oct.
Publication Type	Journal Article.

Result 42.

Unique Identifier	28707495
Authors	Carlin BW; Schuldheisz SK; Noth I; Criner GJ.
Authors Full Name	Carlin, Brian W; Schuldheisz, Sandra K; Noth, Imre; Criner, Gerard J.
Title	Individualizing the selection of long-acting bronchodilator therapy for patients with COPD: considerations in primary care.
Source	Postgraduate Medicine. 129(7):725-733, 2017 Sep.
Publication Type	Journal Article.

Result 43.

Unique Identifier	28542120
Authors	Taylor CA; Greenlund SF; McGuire LC; Lu H; Croft JB.
Authors Full Name	Taylor, Christopher A; Greenlund, Sujay F; McGuire, Lisa C; Lu, Hua; Croft, Janet B.
Title	Deaths from Alzheimer's Disease - United States, 1999-2014.
Source	MMWR - Morbidity & Mortality Weekly Report. 66(20):521-526, 2017 May 26.
Publication Type	Journal Article.

Result 44.

Unique Identifier	28424546
Authors	Mignani V; Ingravallo F; Mariani E; Chattat R.
Authors Full Name	Mignani, Veronica; Ingravallo, Francesca; Mariani, Elena; Chattat, Rabih.
Title	Perspectives of older people living in long-term care facilities and of their family members toward advance care planning discussions: a systematic review and thematic synthesis. [Review]
Source	Clinical Interventions In Aging. 12:475-484, 2017.
Publication Type	Journal Article. Review.

Result 45.

Unique Identifier	28392683
Authors	Mulpuru S; McKay J; Ronksley PE; Thavorn K; Kobewka DM; Forster AJ.
Authors Full Name	Mulpuru, Sunita; McKay, Jennifer; Ronksley, Paul E; Thavorn, Kednapa; Kobewka, Daniel M; Forster, Alan J.
Title	Factors contributing to high-cost hospital care for patients with COPD.
Source	International Journal of Copd. 12:989-995, 2017.
Publication Type	Journal Article. Observational Study.

Result 46.

Unique Identifier	28292824
Authors	Yim CK; Barron Y; Moore S; Murtaugh C; Lala A; Aldridge M; Goldstein N; Gelfman LP.
Authors Full Name	Yim, Cindi K; Barron, Yolanda; Moore, Stanley; Murtaugh, Chris; Lala, Anuradha; Aldridge, Melissa; Goldstein, Nathan; Gelfman, Laura P.
Title	Hospice Enrollment in Patients With Advanced Heart Failure Decreases Acute Medical Service Utilization.
Source	Circulation: Heart Failure. 10(3), 2017 Mar.
Publication Type	Journal Article. Observational Study.

Result 47.

Unique Identifier	28263377
Authors	Pollack LR; Goldstein NE; Gonzalez WC; Blinderman CD; Maurer MS; Lederer DJ; Baldwin MR.
Authors Full Name	Pollack, Lauren R; Goldstein, Nathan E; Gonzalez, Wendy C; Blinderman, Craig D; Maurer, Mathew S; Lederer, David J; Baldwin, Matthew R.

Title	The Frailty Phenotype and Palliative Care Needs of Older Survivors of Critical Illness.
Source	Journal of the American Geriatrics Society. 65(6):1168-1175, 2017 Jun.
Publication Type	Journal Article.

Result 48.

Unique Identifier	28244346
Authors	Mulqueen K; Coffey A.
Authors Full Name	Mulqueen, Kiri; Coffey, Alice.
Title	Preferences of residents with dementia for end of life care.
Source	Nursing Older People. 29(2):26-30, 2017 Feb 28.
Publication Type	Comparative Study. Journal Article.

Result 49.

Unique Identifier	28209392
Authors	McQuown CM; Frey JA; Amireh A; Chaudhary A.
Authors Full Name	McQuown, Colleen M; Frey, Jennifer A; Amireh, Ahmad; Chaudhary, Ali.
Title	Transfer of DNR orders to the ED from extended care facilities.
Source	American Journal of Emergency Medicine. 35(7):983-985, 2017 Jul.
Publication Type	Journal Article.

Result 50.

Unique Identifier	28143412
Authors	Ploeg J; Matthew-Maich N; Fraser K; Dufour S; McAiney C; Kaasalainen S; Markle-Reid M; Upshur R; Cleghorn L; Emili A.
Authors Full Name	Ploeg, Jenny; Matthew-Maich, Nancy; Fraser, Kimberly; Dufour, Sinead; McAiney, Carrie; Kaasalainen, Sharon; Markle-Reid, Maureen; Upshur, Ross; Cleghorn, Laura; Emili, Anna.
Title	Managing multiple chronic conditions in the community: a Canadian qualitative study of the experiences of older adults, family caregivers and healthcare providers.
Source	BMC Geriatrics. 17(1):40, 2017 Jan 31.
Publication Type	Journal Article.

Result 51.

Unique Identifier	26656032
Authors	Pouliot K; Weisse CS; Pratt DS; DiSorbo P.
Authors Full Name	Pouliot, Katherine; Weisse, Carol S; Pratt, David S; DiSorbo, Philip.
Title	First-Year Analysis of a New, Home-Based Palliative Care Program Offered Jointly by a Community Hospital and Local Visiting Nurse Service.
Source	American Journal of Hospice & Palliative Medicine. 34(2):166-172, 2017 Mar.
Publication Type	Journal Article.

Result 52.

Unique Identifier	26494830
Authors	Manu ER; Mody L; McNamara SE; Vitale CA.
Authors Full Name	Manu, Erika R; Mody, Lona; McNamara, Sara E; Vitale, Caroline A.
Title	Advance Directives and Care Received by Older Nursing Home Residents.
Source	American Journal of Hospice & Palliative Medicine. 34(2):105-110, 2017 Mar.
Publication Type	Journal Article.

Result 53.

Unique Identifier	25168076
Authors	Elliott M; Nicholson C.
Authors Full Name	Elliott, Margaret; Nicholson, Caroline.
Title	A qualitative study exploring use of the surprise question in the care of older people: perceptions of general practitioners and challenges for practice.

Source	BMJ supportive & palliative care. 7(1):32-38, 2017 Mar.
Publication Type	Journal Article.

Result 54.

Unique Identifier	27883315
Authors	Stryckers M; Nagler EV; Van Biesen W.
Authors Full Name	Stryckers, Marijke; Nagler, Evi V; Van Biesen, Wim.
Title	The Need for Accurate Risk Prediction Models for Road Mapping, Shared Decision Making and Care Planning for the Elderly with Advanced Chronic Kidney Disease.
Source	Prilozi Makedonska Akademija Na Naukite I Umetnostite Oddelenie Za Medicinski Nauki. 37(2-3):33-42, 2016 Nov 01.
Publication Type	Journal Article.

Result 55.

Unique Identifier	27822976
Authors	Winthereik A; Neergaard M; Vedsted P; Jensen A.
Authors Full Name	Winthereik, Anna; Neergaard, Mette; Vedsted, Peter; Jensen, Anders.
Title	Danish general practitioners' self-reported competences in end-of-life care.
Source	Scandinavian Journal of Primary Health Care. 34(4):420-427, 2016 Dec.
Publication Type	Journal Article.

Result 56.

Unique Identifier	27381487
Authors	Fisher RF; Lasserson D; Hayward G.
Authors Full Name	Fisher, Rebecca Fr; Lasserson, Daniel; Hayward, Gail.
Title	Out-of-hours primary care use at the end of life: a descriptive study.
Source	British Journal of General Practice. 66(650):e654-60, 2016 Sep.
Publication Type	Journal Article.

Result 57.

Unique Identifier	27257779
Authors	Casarett D; Teno J.
Authors Full Name	Casarett, David; Teno, Joan.
Title	Why Population Health and Palliative Care Need Each Other.
Source	JAMA. 316(1):27-8, 2016 Jul 05.
Publication Type	Journal Article.

Result 58.

Unique Identifier	27251335
Authors	Marcucci FC; Cabrera MA; Perilla AB; Brun MM; de Barros EM; Martins VM; Rosenberg JP; Yates P.
Authors Full Name	Marcucci, Fernando C I; Cabrera, Marcos A S; Perilla, Anamaria Baquero; Brun, Marilia Maroneze; de Barros, Eder Marcos L; Martins, Vanessa M; Rosenberg, John P; Yates, Patsy.
Title	Identification and characteristics of patients with palliative care needs in Brazilian primary care.
Source	BMC Palliative Care. 15:51, 2016 Jun 01.
Publication Type	Journal Article.

Result 59.

Unique Identifier	27215058
Authors	Wasylynuk BA; Davison SN.
Authors Full Name	Wasylynuk, Betty Ann; Davison, Sara N.
Title	An overview of advance care planning for patients with advanced chronic kidney disease: The basics.

Source Cannt Journal. 26(1):24-9, 2016 Jan-Mar.

Publication Type Journal Article.

Result 60.

Unique Identifier 27139373

Authors Bailey C; Hewison A; Karasouli E; Staniszewska S; Munday D.

Authors Full Name Bailey, Cara; Hewison, Alistair; Karasouli, Eleni; Staniszewska, Sophie; Munday, Daniel.

Title Hospital care following emergency admission: a critical incident case study of the experiences of patients with advanced lung cancer and Chronic Obstructive Pulmonary Disease.

Source Journal of Clinical Nursing. 25(15-16):2168-79, 2016 Aug.

Publication Type Journal Article.

Result 61.

Unique Identifier 27113154

Authors Pak E; Wald J; Kirkpatrick JN.

Authors Full Name Pak, Esther; Wald, Joyce; Kirkpatrick, James N.

Title Multimorbidity and End of Life Care in Patients with Cardiovascular Disease. [Review]

Source Clinics in Geriatric Medicine. 32(2):385-97, 2016 May.

Publication Type Journal Article. Review.

Result 62.

Unique Identifier 27079676

Authors Hopp FP; Zalenski RJ; Waselewsky D; Burn J; Camp J; Welch RD; Levy P.

Authors Full Name Hopp, Faith P; Zalenski, Robert J; Waselewsky, Denise; Burn, Jeri; Camp, Jessica; Welch, Robert D; Levy, Phillip.

Title Results of a Hospital-Based Palliative Care Intervention for Patients With an Acute Exacerbation of Chronic Heart Failure.

Source Journal of Cardiac Failure. 22(12):1033-1036, 2016 Dec.

Publication Type Journal Article. Randomized Controlled Trial.

Result 63.

Unique Identifier 27079335

Authors Rich MW; Chyun DA; Skolnick AH; Alexander KP; Forman DE; Kitzman DW; Maurer MS; McClurken JB; Resnick BM; Shen WK; Tirschwell DL; American Heart Association Older Populations Committee of the Council on Clinical Cardiology, Council on Cardiovascular and Stroke Nursing, Council on Cardiovascular Surgery and Anesthesia, and Stroke Council; American College of Cardiology; and American Geriatrics Society.

Authors Full Name Rich, Michael W; Chyun, Deborah A; Skolnick, Adam H; Alexander, Karen P; Forman, Daniel E; Kitzman, Dalane W; Maurer, Mathew S; McClurken, James B; Resnick, Barbara M; Shen, Win K; Tirschwell, David L; American Heart Association Older Populations Committee of the Council on Clinical Cardiology, Council on Cardiovascular and Stroke Nursing, Council on Cardiovascular Surgery and Anesthesia, and Stroke Council; American College of Cardiology; and American Geriatrics Society.

Title Knowledge Gaps in Cardiovascular Care of the Older Adult Population: A Scientific Statement From the American Heart Association, American College of Cardiology, and American Geriatrics Society.

Source Journal of the American College of Cardiology. 67(20):2419-2440, 2016 May 24.

Publication Type Journal Article.

Result 64.

Unique Identifier 27053406

Authors Slomka J; Prince-Paul M; Webel A; Daly BJ.

Authors Full Name Slomka, Jacquelyn; Prince-Paul, Maryjo; Webel, Allison; Daly, Barbara J.

Title Palliative Care, Hospice, and Advance Care Planning: Views of People Living with HIV and Other Chronic Conditions.

Source Journal of the Association of Nurses in AIDS Care. 27(4):476-84, 2016 Jul-Aug.

Publication Type Journal Article. Research Support, N.I.H., Extramural.

Result 65.

Unique Identifier 27051994

Authors	Reymond L; Cooper K; Parker D; Chapman M.
Authors Full Name	Reymond, Liz; Cooper, Karen; Parker, Deborah; Chapman, Michael.
Title	End-of-life care: Proactive clinical management of older Australians in the community.
Source	Australian Family Physician. 45(1):76-8, 2016 Jan-Feb.
Publication Type	Journal Article.

Result 66.

Unique Identifier	26825873
Authors	Chao CT; Tsai HB; Shih CY; Hsu SH; Hung YC; Lai CF; Ueng RH; Chan DC; Hwang JJ; Huang SJ.
Authors Full Name	Chao, Chia-Ter; Tsai, Hung-Bin; Shih, Chih-Yuan; Hsu, Su-Hsuan; Hung, Yu-Chien; Lai, Chun-Fu; Ueng, Ruey-Hsiuang; Chan, Ding-Cheng; Hwang, Juey-Jen; Huang, Sheng-Jean.
Title	Establishment of a renal supportive care program: Experience from a rural community hospital in Taiwan. [Review]
Source	Journal of the Formosan Medical Association. 115(7):490-500, 2016 Jul.
Publication Type	Journal Article. Review.

Result 67.

Unique Identifier	26603186
Authors	Sahlen KG; Boman K; Brannstrom M.
Authors Full Name	Sahlen, Klas-Goran; Boman, Kurt; Brannstrom, Margareta.
Title	A cost-effectiveness study of person-centered integrated heart failure and palliative home care: Based on a randomized controlled trial.
Source	Palliative Medicine. 30(3):296-302, 2016 Mar.
Publication Type	Journal Article. Randomized Controlled Trial. Research Support, Non-U.S. Gov't.

Result 68.

Unique Identifier	26373835
Authors	Aggarwal SK; Ghosh A; Cheng MJ; Luton K; Lowet PF; Berger A.
Authors Full Name	Aggarwal, Sunil K; Ghosh, Amrita; Cheng, M Jennifer; Luton, Kathleen; Lowet, Peter F; Berger, Ann.
Title	Initiating pain and palliative care outpatient services for the suburban underserved in Montgomery County, Maryland: Lessons learned at the NIH Clinical Center and MobileMed.
Source	Palliative & Supportive Care. 14(4):381-6, 2016 08.
Publication Type	Journal Article.

Result 69.

Unique Identifier	26261374
Authors	Kwok AO; Yuen SK; Yong DS; Tse DM.
Authors Full Name	Kwok, Annie O; Yuen, Sze-Kit; Yong, David S; Tse, Doris M.
Title	The Symptoms Prevalence, Medical Interventions, and Health Care Service Needs for Patients With End-Stage Renal Disease in a Renal Palliative Care Program.
Source	American Journal of Hospice & Palliative Medicine. 33(10):952-958, 2016 Dec.
Publication Type	Journal Article.

Result 70.

Unique Identifier	25818406
Authors	Watson E; Shinkins B; Frith E; Neal D; Hamdy F; Walter F; Weller D; Wilkinson C; Faithfull S; Wolstenholme J; Sooriakumaran P; Kastner C; Campbell C; Neal R; Butcher H; Matthews M; Perera R; Rose P.
Authors Full Name	Watson, Eila; Shinkins, Bethany; Frith, Emma; Neal, David; Hamdy, Freddie; Walter, Fiona; Weller, David; Wilkinson, Clare; Faithfull, Sara; Wolstenholme, Jane; Sooriakumaran, Prasanna; Kastner, Christof; Campbell, Christine; Neal, Richard; Butcher, Hugh; Matthews, Mike; Perera, Rafael; Rose, Peter.
Title	Symptoms, unmet needs, psychological well-being and health status in survivors of prostate cancer: implications for redesigning follow-up.
Source	BJU International. 117(6B):E10-9, 2016 Jun.
Publication Type	Journal Article.

Result 71.

Unique Identifier	25743437
Authors	Magee C; Koffman J.
Authors Full Name	Magee, Claire; Koffman, Jonathan.
Title	Out-of-hours palliative care: what are the educational needs and preferences of general practitioners?.
Source	BMJ supportive & palliative care. 6(3):362-8, 2016 Sep.
Publication Type	Journal Article.

Result 72.

Unique Identifier	25023218
Authors	Mason B; Nanton V; Epiphaniou E; Murray SA; Donaldson A; Shipman C; Daveson BA; Harding R; Higginson IJ; Munday D; Barclay S; Dale J; Kendall M; Worth A; Boyd K.
Authors Full Name	Mason, Bruce; Nanton, Veronica; Epiphaniou, Eleni; Murray, Scott A; Donaldson, Anne; Shipman, Cathy; Daveson, Barbara A; Harding, Richard; Higginson, Irene J; Munday, Dan; Barclay, Stephen; Dale, Jeremy; Kendall, Marilyn; Worth, Allison; Boyd, Kirsty.
Title	'My body's falling apart.' Understanding the experiences of patients with advanced multimorbidity to improve care: serial interviews with patients and carers.
Source	BMJ supportive & palliative care. 6(1):60-5, 2016 Mar.
Publication Type	Journal Article. Multicenter Study. Research Support, Non-U.S. Gov't.

Result 73.

Unique Identifier	22663083
Authors	Kim Y; Yen IH; Rabow MW.
Authors Full Name	Kim, Yunie; Yen, Irene H; Rabow, Michael W.
Title	Comparing Symptom Burden in Patients with Metastatic and Nonmetastatic Cancer.
Source	Journal of Palliative Medicine. 19(1):64-8, 2016 Jan.
Publication Type	Comparative Study. Journal Article.

Result 74.

Unique Identifier	26897863
Authors	Thavarajah N; Menjak I; Trudeau M; Mehta R; Wright F; Leahey A; Ellis J; Gallagher D; Moore J; Bristow B; Kay N; Szumacher E.
Authors Full Name	Thavarajah, Nemica; Menjak, Ines; Trudeau, Maureen; Mehta, Rajin; Wright, Frances; Leahey, Angela; Ellis, Janet; Gallagher, Damian; Moore, Jennifer; Bristow, Bonnie; Kay, Noreen; Szumacher, Ewa.
Title	Towards an optimal multidisciplinary approach to breast cancer treatment for older women. [Review]
Source	Canadian Oncology Nursing Journal. 25(4):384-408, 2015.
Publication Type	Journal Article. Review.

Result 75.

Unique Identifier	26496646
Authors	Morgan DG; Kosteniuk JG; Stewart NJ; O'Connell ME; Kirk A; Crossley M; Dal Bello-Haas V; Forbes D; Innes A.
Authors Full Name	Morgan, Debra G; Kosteniuk, Julie G; Stewart, Norma J; O'Connell, Megan E; Kirk, Andrew; Crossley, Margaret; Dal BelloHaas, Vanina; Forbes, Dorothy; Innes, Anthea.
Title	Availability and Primary Health Care Orientation of Dementia-Related Services in Rural Saskatchewan, Canada.
Source	Home Health Care Services Quarterly. 34(3-4):137-58, 2015.
Publication Type	Journal Article. Research Support, Non-U.S. Gov't.

Result 76.

Unique Identifier	26480973
Authors	Shaulov A; Frankel M; Rubinow A; Maaravi Y; Brezis M.
Authors Full Name	Shaulov, Adir; Frankel, Meir; Rubinow, Alan; Maaravi, Yoram; Brezis, Mayer.
Title	Preparedness for End of Life-a Survey of Jerusalem District Nursing Homes.
Source	Journal of the American Geriatrics Society. 63(10):2114-9, 2015 Oct.
Publication Type	Journal Article.

Result 77.

Unique Identifier 26429705

Authors Hedinger D; Hammig O; Bopp M; Swiss National Cohort Study Group.

Authors Full Name Hedinger, Damian; Hammig, Oliver; Bopp, Matthias; Swiss National Cohort Study Group.

Title Social determinants of duration of last nursing home stay at the end of life in Switzerland: a retrospective cohort study.

Source BMC Geriatrics. 15:114, 2015 Oct 01.

Publication Type Journal Article. Research Support, Non-U.S. Gov't.

Result 78.

Unique Identifier 26398744

Authors Burge F; Lawson B; Johnston G; Asada Y; McIntyre PF; Flowerdew G.

Authors Full Name Burge, Fred; Lawson, Beverley; Johnston, Grace; Asada, Yukiko; McIntyre, Paul F; Flowerdew, Gordon.

Title Preferred and Actual Location of Death: What Factors Enable a Preferred Home Death?.

Source Journal of Palliative Medicine. 18(12):1054-9, 2015 Dec.

Publication Type Journal Article. Research Support, Non-U.S. Gov't.

Result 79.

Unique Identifier 26249645

Authors Pesut B; McLean T; Reimer-Kirkham S; Hartrick-Doane G; Hutchings D; Russell LB.

Authors Full Name Pesut, Barbara; McLean, Tammy; Reimer-Kirkham, Sheryl; Hartrick-Doane, Gweneth; Hutchings, Deanna; Russell, Lara B.

Title Educating registered nursing and healthcare assistant students in community-based supportive care of older adults: A mixed methods study.

Source Nurse Education Today. 35(9):e90-6, 2015 Sep.

Publication Type Journal Article. Research Support, Non-U.S. Gov't.

Result 80.

Unique Identifier 26209094

Authors Stiel S; Heckel M; Seifert A; Frauendorf T; Hanke RM; Ostgathe C.

Authors Full Name Stiel, Stephanie; Heckel, Maria; Seifert, Andreas; Frauendorf, Tobias; Hanke, Roland Martin; Ostgathe, Christoph.

Title Comparison of terminally ill cancer- vs. non-cancer patients in specialized palliative home care in Germany - a single service analysis.

Source BMC Palliative Care. 14:34, 2015 Jul 25.

Publication Type Journal Article.

Result 81.

Unique Identifier 26084951

Authors Cloyes KG; Berry PH; Martz K; Supiano K.

Authors Full Name Cloyes, Kristin G; Berry, Patricia H; Martz, Kim; Supiano, Katherine.

Title Characteristics of Prison Hospice Patients: Medical History, Hospice Care, and End-of-Life Symptom Prevalence.

Source Journal of Correctional Health Care. 21(3):298-308, 2015 Jul.

Publication Type Journal Article. Research Support, Non-U.S. Gov't.

Result 82.

Unique Identifier 26066323

Authors Eneanya ND; Goff SL; Martinez T; Gutierrez N; Klingensmith J; Griffith JL; Garvey C; Kitsen J; Germain MJ; Marr L; Berzoff J; Unruh M; Cohen LM.

Authors Full Name Eneanya, Nwamaka D; Goff, Sarah L; Martinez, Talaya; Gutierrez, Natalie; Klingensmith, Jamie; Griffith, John L; Garvey, Casey; Kitsen, Jenny; Germain, Michael J; Marr, Lisa; Berzoff, Joan; Unruh, Mark; Cohen, Lewis M.

Title Shared decision-making in end-stage renal disease: a protocol for a multi-center study of a communication intervention to improve end-of-life care for dialysis patients.

Source	BMC Palliative Care. 14:30, 2015 Jun 12.
Publication Type	Clinical Trial. Journal Article. Multicenter Study. Research Support, Non-U.S. Gov't.

Result 83.

Unique Identifier	26047831
Authors	Nur U; Quaresma M; De Stavola B; Peake M; Rachet B.
Authors Full Name	Nur, Ula; Quaresma, Manuela; De Stavola, Bianca; Peake, Michael; Rachet, Bernard.
Title	Inequalities in non-small cell lung cancer treatment and mortality.
Source	Journal of Epidemiology & Community Health. 69(10):985-92, 2015 Oct.
Publication Type	Journal Article. Research Support, Non-U.S. Gov't.

Result 84.

Unique Identifier	26028347
Authors	Buckingham S; Kendall M; Ferguson S; MacNee W; Sheikh A; White P; Worth A; Boyd K; Murray SA; Pinnock H.
Authors Full Name	Buckingham, Susan; Kendall, Marilyn; Ferguson, Susie; MacNee, William; Sheikh, Aziz; White, Patrick; Worth, Allison; Boyd, Kirsty; Murray, Scott A; Pinnock, Hilary.
Title	HELPing older people with very severe chronic obstructive pulmonary disease (HELP-COPD): mixed-method feasibility pilot randomised controlled trial of a novel intervention.
Source	NPJ Primary Care Respiratory Medicine. 25:15020, 2015 Apr 16.
Publication Type	Journal Article. Randomized Controlled Trial. Research Support, Non-U.S. Gov't.

Result 85.

Unique Identifier	25939666
Authors	Pesut B; Hooper BP; Robinson CA; Bottorff JL; Sawatzky R; Dalhuisen M.
Authors Full Name	Pesut, B; Hooper, B P; Robinson, C A; Bottorff, J L; Sawatzky, R; Dalhuisen, M.
Title	Feasibility of a rural palliative supportive service.
Source	Rural & Remote Health. 15(2):3116, 2015 Apr-Jun.
Publication Type	Journal Article. Multicenter Study. Research Support, Non-U.S. Gov't.

Result 86.

Unique Identifier	25828558
Authors	Kendall M; Carduff E; Lloyd A; Kimbell B; Cavers D; Buckingham S; Boyd K; Grant L; Worth A; Pinnock H; Sheikh A; Murray SA.
Authors Full Name	Kendall, Marilyn; Carduff, Emma; Lloyd, Anna; Kimbell, Barbara; Cavers, Debbie; Buckingham, Susan; Boyd, Kirsty; Grant, Liz; Worth, Allison; Pinnock, Hilary; Sheikh, Aziz; Murray, Scott A.
Title	Different Experiences and Goals in Different Advanced Diseases: Comparing Serial Interviews With Patients With Cancer, Organ Failure, or Frailty and Their Family and Professional Carers.
Source	Journal of Pain & Symptom Management. 50(2):216-24, 2015 Aug.
Publication Type	Comparative Study. Journal Article. Research Support, Non-U.S. Gov't.

Result 87.

Unique Identifier	25680737
Authors	Goff SL; Eneanya ND; Feinberg R; Germain MJ; Marr L; Berzoff J; Cohen LM; Unruh M.
Authors Full Name	Goff, Sarah L; Eneanya, Nwamaka D; Feinberg, Rebecca; Germain, Michael J; Marr, Lisa; Berzoff, Joan; Cohen, Lewis M; Unruh, Mark.
Title	Advance care planning: a qualitative study of dialysis patients and families.
Source	Clinical Journal of The American Society of Nephrology: CJASN. 10(3):390-400, 2015 Mar 06.
Publication Type	Journal Article. Research Support, N.I.H., Extramural. Research Support, Non-U.S. Gov't.

Result 88.

Unique Identifier	25637404
Authors	Silva C; Ramalho C; Luz I; Monteiro J; Fresco P.
Authors Full Name	Silva, Cristina; Ramalho, Celia; Luz, Isabel; Monteiro, Joaquim; Fresco, Paula.
Title	Drug-related problems in institutionalized, polymedicated elderly patients: opportunities for pharmacist intervention.

Source International Journal of Clinical Pharmacy. 37(2):327-34, 2015 Apr.

Publication Type Journal Article. Observational Study. Research Support, Non-U.S. Gov't.

Result 89.

Unique Identifier	25561640
Authors	Leff B; Carlson CM; Saliba D; Ritchie C.
Authors Full Name	Leff, Bruce; Carlson, Charlotte M; Saliba, Debra; Ritchie, Christine.
Title	The invisible homebound: setting quality-of-care standards for home-based primary and palliative care.
Source	Health Affairs. 34(1):21-9, 2015 Jan.
Publication Type	Journal Article. Research Support, Non-U.S. Gov't.

Result 90.

Unique Identifier	25554484
Authors	Kim MT; Kim KB; Han HR; Huh B; Nguyen T; Lee HB.
Authors Full Name	Kim, Miyong T; Kim, Kim B; Han, Hae-Ra; Huh, Boyun; Nguyen, Tam; Lee, Hochang B.
Title	Prevalence and Predictors of Depression in Korean American Elderly: Findings from the Memory and Aging Study of Koreans (MASK).
Source	American Journal of Geriatric Psychiatry. 23(7):671-83, 2015 Jul.
Publication Type	Journal Article. Research Support, N.I.H., Extramural. Research Support, Non-U.S. Gov't.

Result 91.

Unique Identifier	25547480
Authors	Washington KT; Pike KC; Demiris G; Oliver DP.
Authors Full Name	Washington, Karla T; Pike, Kenneth C; Demiris, George; Oliver, Debra Parker.
Title	Unique characteristics of informal hospice cancer caregiving.
Source	Supportive Care in Cancer. 23(7):2121-8, 2015 Jul.
Publication Type	Journal Article. Randomized Controlled Trial. Research Support, N.I.H., Extramural.

Result 92.

Unique Identifier	25544001
Authors	Reyniers T; Deliens L; Pasman HR; Morin L; Addington-Hall J; Frova L; Cardenas-Turanzas M; Onwuteaka-Philipsen B; Naylor W; Ruiz-Ramos M; Wilson DM; Loucka M; Csikos A; Rhee YJ; Teno J; Cohen J; Houttekier D.
Authors Full Name	Reyniers, Thijs; Deliens, Luc; Pasman, H Roeline; Morin, Lucas; Addington-Hall, Julia; Frova, Luisa; Cardenas-Turanzas, Marylou; Onwuteaka-Philipsen, Bregje; Naylor, Wayne; Ruiz-Ramos, Miguel; Wilson, Donna M; Loucka, Martin; Csikos, Agnes; Rhee, Yong Joo; Teno, Joan; Cohen, Joachim; Houttekier, Dirk.
Title	International variation in place of death of older people who died from dementia in 14 European and non-European countries.
Source	Journal of the American Medical Directors Association. 16(2):165-71, 2015 Feb.
Publication Type	Comparative Study. Journal Article. Research Support, Non-U.S. Gov't.

Result 93.

Unique Identifier	25205231
Authors	Zimmerman S; Cohen L; van der Steen JT; Reed D; van Soest-Poortvliet MC; Hanson LC; Sloane PD.
Authors Full Name	Zimmerman, Sheryl; Cohen, Lauren; van der Steen, Jenny T; Reed, David; van Soest-Poortvliet, Mirjam C; Hanson, Laura C; Sloane, Philip D.
Title	Measuring end-of-life care and outcomes in residential care/assisted living and nursing homes.
Source	Journal of Pain & Symptom Management. 49(4):666-79, 2015 Apr.
Publication Type	Journal Article. Multicenter Study. Randomized Controlled Trial. Research Support, Non-U.S. Gov't.

Result 94.

Unique Identifier	24863394
Authors	Bing-Jonsson PC; Bjork IT; Hofoss D; Kirkevold M; Foss C.

Authors Full Name	Bing-Jonsson, Pia Cecilie; Bjork, Ida Torunn; Hofoss, Dag; Kirkevold, Marit; Foss, Christina.
Title	Competence in advanced older people nursing: development of 'nursing older people--competence evaluation tool'.
Source	International Journal of Older People Nursing. 10(1):59-72, 2015 Mar.
Publication Type	Journal Article.

Result 95.

Unique Identifier	24644201
Authors	Girgis A; Abernethy AP; Currow DC.
Authors Full Name	Girgis, Afaf; Abernethy, Amy P; Currow, David C.
Title	Caring at the end of life: do cancer caregivers differ from other caregivers?.
Source	BMJ supportive & palliative care. 5(5):513-7, 2015 Dec.
Publication Type	Journal Article. Research Support, Non-U.S. Gov't.

Result 96.

Unique Identifier	25927972
Authors	Calanzani N; Moens K; Cohen J; Higginson IJ; Harding R; Deliens L; Toscani F; Ferreira PL; Bausewein C; Daveson BA; Gysels M; Ceulemans L; Gomes B; Project PRISMA.
Authors Full Name	Calanzani, Natalia; Moens, Katrien; Cohen, Joachim; Higginson, Irene J; Harding, Richard; Deliens, Luc; Toscani, Franco; Ferreira, Pedro L; Bausewein, Claudia; Daveson, Barbara A; Gysels, Marjolein; Ceulemans, Lucas; Gomes, Barbara; Project PRISMA.
Title	Choosing care homes as the least preferred place to die: a cross-national survey of public preferences in seven European countries.
Source	BMC Palliative Care. 13:48, 2014 Oct 23.
Publication Type	Journal Article. Research Support, Non-U.S. Gov't.

Result 97.

Unique Identifier	25473275
Authors	Ellis G; Marshall T; Ritchie C.
Authors Full Name	Ellis, Graham; Marshall, Trudi; Ritchie, Claire.
Title	Comprehensive geriatric assessment in the emergency department. [Review]
Source	Clinical Interventions In Aging. 9:2033-43, 2014.
Publication Type	Journal Article. Review.

Result 98.

Unique Identifier	25376084
Authors	Tschirhart EC; Du Q; Kelley AS.
Authors Full Name	Tschirhart, Evan C; Du, Qingling; Kelley, Amy S.
Title	Factors influencing the use of intensive procedures at the end of life.
Source	Journal of the American Geriatrics Society. 62(11):2088-94, 2014 Nov.
Publication Type	Journal Article. Research Support, N.I.H., Extramural. Research Support, Non-U.S. Gov't.

Result 99.

Unique Identifier	25375799
Authors	Kerr CW; Donohue KA; Tangeman JC; Serehali AM; Knodel SM; Grant PC; Luczkiewicz DL; Mylotte K; Marien MJ.
Authors Full Name	Kerr, Christopher W; Donohue, Kathleen A; Tangeman, John C; Serehali, Amin M; Knodel, Sarah M; Grant, Pei C; Luczkiewicz, Debra L; Mylotte, Kathleen; Marien, Melanie J.
Title	Cost savings and enhanced hospice enrollment with a home-based palliative care program implemented as a hospice-private payer partnership.
Source	Journal of Palliative Medicine. 17(12):1328-35, 2014 Dec.
Publication Type	Comparative Study. Journal Article. Observational Study.

Result 100.

Unique Identifier	25313997
Authors	Herce ME; Elmore SN; Kalanga N; Keck JW; Wroe EB; Phiri A; Mayfield A; Chingoli F; Beste JA; Tengatenga L; Bazile J; Krakauer EL; Rigodon J.
Authors Full Name	Herce, Michael E; Elmore, Shekinah N; Kalanga, Noel; Keck, James W; Wroe, Emily B; Phiri, Atupere; Mayfield, Alishya; Chingoli, Felix; Beste, Jason A; Tengatenga, Listern; Bazile, Junior; Krakauer, Eric L; Rigodon, Jonas.
Title	Assessing and responding to palliative care needs in rural sub-Saharan Africa: results from a model intervention and situation analysis in Malawi.
Source	PLoS ONE [Electronic Resource]. 9(10):e110457, 2014.
Publication Type	Journal Article. Research Support, N.I.H., Extramural. Research Support, Non-U.S. Gov't.
