# Supplementary material for: Addressing inequity in palliative care provision for older people living with multimorbidity. Perspectives of community-dwelling older people on their palliative care needs: A scoping review
Source: Palliat Med. 2022 Aug 24;37(4):475–97. doi: 10.1177/02692163221118230 (PMC10074761; doi:10.1177/02692163221118230)
Supplement: sj-pdf-3-pmj-10.1177_02692163221118230 – Supplemental material for Addressing inequity in palliative care provision for older people living with multimorbidity. Perspectives of community-dwelling older people on their palliative care needs: A scoping review [file sj-pdf-3-pmj-10.1177_02692163221118230.pdf]

**Appendix 3: Mixed Methods Appraisal Tool Reporting for Included Papers**

| Reference | RQ (research question) | | Qualitative | | | | | Quantitative RCT | | | | | Quantitative Non-RCT | | | | | Quantitative Descriptive | | | | | Mixed Methods | | | | |
|---|---|---|---|---|---|---|---|---|---|---|---|---|---|---|---|---|---|---|---|---|---|---|---|---|---|---|---|
| | Clear RQ | Objectives | 1.1 | 1.2 | 1.3 | 1.4 | 1.5 | 2.1 | 2.2 | 2.3 | 2.4 | 2.5 | 3.1 | 3.2 | 3.3 | 3.4 | 3.5 | 4.1 | 4.2 | 4.3 | 4.4 | 4.5 | 5.1 | 5.2 | 5.3 | 5.4 | 5.5 |
| Amblas-Novellas (2016) | Y | Y | | | | | | | | | | | U | Y | N | Y | Y | | | | | | | | | | |
| Beach (2018) | Y | Y | | | | | | | | | | | | | | | | Y | Y | Y | N | Y | | | | | |
| Bone (2019) | Y | Y | | | | | | | | | | | | | | | | U | Y | Y | Y | Y | | | | | |
| Brandt (2005) | Y | Y | | | | | | | | | | | Y | Y | U | U | Y | | | | | | | | | | |
| Chan (2007) | Y | Y | | | | | | | | | | | Y | Y | Y | N | Y | | | | | | | | | | |
| Chochinov (2016) | Y | Y | | | | | | | | | | | | | | | | Y | Y | Y | Y | Y | | | | | |
| Gomez-Batiste (2014) | Y | Y | | | | | | | | | | | Y | Y | U | Y | Y | | | | | | | | | | |
| Goodridge (2005) | Y | Y | Y | Y | Y | Y | Y | | | | | | | | | | | | | | | | | | | | |
| Kayser-Jones (2002) | Y | Y | Y | Y | U | Y | Y | | | | | | | | | | | | | | | | | | | | |
| Kayser-Jones et al (2003) | Y | Y | Y | Y | Y | Y | Y | | | | | | | | | | | | | | | | | | | | |
| Kendall (2015) | Y | Y | Y | Y | Y | Y | Y | | | | | | | | | | | | | | | | | | | | |
| Koslov (2018) | Y | Y | | | | | | | | | | | Y | Y | Y | Y | U | | | | | | | | | | |
| Kramer (2013) | Y | Y | Y | Y | Y | Y | Y | | | | | | | | | | | | | | | | | | | | |
| Kricke (2011) | Y | Y | | | | | | | | | | | Y | Y | Y | Y | Y | | | | | | | | | | |
| Lambotte (2018) | Y | Y | | | | | | | | | | | Y | Y | Y | Y | Y | | | | | | | | | | |
| Lee (2019) | Y | Y | | | | | | | | | | | | | | | | Y | Y | Y | Y | Y | | | | | |
| Marcucci (2016) | Y | Y | | | | | | | | | | | Y | Y | Y | Y | Y | | | | | | | | | | |
| Mason (2016) | Y | Y | | | | | | | | | | | Y | Y | U | Y | Y | | | | | | | | | | |
| McVey (2013) | Y | Y | Y | Y | Y | Y | Y | | | | | | | | | | | | | | | | | | | | |
| Nicholson (2018) | Y | Y | | | | | | | | | | | Y | Y | N | Y | Y | | | | | | | | | | |
| Osterlind (2016) | Y | Y | Y | Y | Y | Y | Y | | | | | | | | | | | | | | | | | | | | |
| Parker (2005) | Y | Y | | | | | | | | | | | | | | | | | | | | | Y | Y | Y | U | U |
| Reinke (2019 | Y | Y | | | | | | | | | | | Y | Y | Y | Y | Y | | | | | | | | | | |
| Sloane (2013) | Y | Y | | | | | | | | | | | | | | | | Y | Y | Y | Y | Y | | | | | |
| Strohbuecker | Y | Y | Y | Y | Y | Y | Y | | | | | | | | | | | | | | | | | | | | |
| Van den Brink (2018) | Y | Y | | | | | | | | | | | Y | Y | Y | Y | Y | | | | | | | | | | |
| Vandenburg (2005) | Y | Y | | | | | | | | | | | | | | | | Y | Y | Y | U | Y | | | | | |
| Vohra (2006) | Y | Y | | | | | | | | | | | | | | | | Y | U | U | U | U | | | | | |

**MMAT Key**
Y: Yes;
N: No;
U: Unclear

**Reporting Notes**

- Scoring is not recommended for the MMAT, this table is indicative of the quality of included studies.
- Studies were appraised if they used a self-defined study design – case study/ cross-sectional and best fit MMAT criteria/ description.
- Studies were appraised according to reported data in the paper and not data reported in earlier/linked publications.
